# Iron homeostasis governs erythroid phenotype in Polycythemia Vera

**DOI:** 10.1182/blood.2022016779

**Published:** 2023-06-29

**Authors:** Cavan Bennett, Victoria E Jackson, Anne Pettikiriarachchi, Thomas Hayman, Ute Schaeper, Gemma Moir-Meyer, Katherine Fielding, Ricardo Ataide, Danielle Clucas, Andrew Baldi, Alexandra L Garnham, Connie SN Li-Wai-Suen, Stephen J Loughran, E Joanna Baxter, Anthony R Green, Warren S Alexander, Melanie Bahlo, Kate Burbury, Ashley P Ng, Sant-Rayn Pasricha

**Affiliations:** 1 Population Health and Immunity Division, The Walter and Eliza Hall Institute of Medical Research, Parkville, Victoria, Australia; 2 Department of Medical Biology, University of Melbourne, Parkville, Victoria, Australia; 3 Silence Therapeutics GmbH, Berlin, Germany; 4 Department of Infectious Diseases, Peter Doherty Institute, University of Melbourne, Parkville, Victoria, Australia; 5 Diagnostic Haematology, The Royal Melbourne Hospital, Parkville, Victoria, Australia; 6 Bioinformatics Division, The Walter and Eliza Hall Institute of Medical Research, Parkville, Victoria, Australia; 7 Wellcome–MRC Cambridge Stem Cell Institute, Jeffrey Cheah Biomedical Centre, University of Cambridge, Cambridge, United Kingdom; 8 Department of Haematology, University of Cambridge, Cambridge, United Kingdom; 9 Blood Cells and Blood Cancer Division, The Walter and Eliza Hall Institute of Medical Research, Parkville, Victoria, Australia; 10 Clinical Haematology at the Peter MacCallum Cancer Centre and The Royal Melbourne Hospital, Melbourne, Victoria, Australia

## Abstract

Polycythemia Vera (PV) is a myeloproliferative neoplasm driven by activating mutations in *JAK2* that result in unrestrained erythrocyte production, increasing patients’ hematocrit and hemoglobin concentration, placing them at risk of life-threatening thrombotic events. Our GWAS of 440 PV cases and 403,351 controls utilizing UK Biobank data found that SNPs in *HFE* known to cause hemochromatosis are highly associated with PV diagnosis, linking iron regulation to PV. Analysis of the FinnGen dataset independently confirmed over-representation of homozygous *HFE* variants in PV patients. HFE influences the expression of hepcidin, the master regulator of systemic iron homeostasis. Through genetic dissection of PV mouse models, we show that the PV erythroid phenotype is directly linked to hepcidin expression: endogenous hepcidin upregulation alleviates erythroid disease whereas hepcidin ablation worsens it. Further, we demonstrate that in PV, hepcidin is not regulated by expanded erythropoiesis but is likely governed by inflammatory cytokines signaling via GP130 coupled receptors. These findings have important implications for understanding the pathophysiology of PV and offer new therapeutic strategies for this disease.

## Introduction

Polycythemia Vera (PV) is a Philadelphia chromosome-negative myeloproliferative neoplasm (MPN) driven by activating mutations in *JAK2*^1–4^ that cause unrestrained erythrocyte production, increasing patients’ hematocrit and hemoglobin concentration. Over 95% of cases harbour the *JAK2* V617F mutation, with the remainder usually exhibiting a mutation in *JAK2* exon 12^5,6^. Complications of elevated hematocrit include venous and arterial thrombosis, and systemic symptoms including headache, visual disturbances, and pruritis^7^. Therapy typically includes regular venesection to maintain hematocrit below 45%^6,8^. This phase of the disease may continue for years before some patients develop fibrotic or leukemic transformation.

Iron availability for erythropoiesis (and other tissues) is governed by hepcidin, the master regulator of systemic iron homeostasis. Hepcidin is produced by the liver and occludes and internalizes the sole cellular iron exporter, ferroportin^9,10^, preventing recycled iron in macrophages and dietary iron absorbed in the intestine from reaching the plasma and hence the bone marrow^11^. Elevated hepcidin thus reduces iron availability, while suppressed hepcidin enhances it^12^. Systemic iron homeostasis is maintained via transcriptional regulation of hepcidin, whereby iron loading upregulates transcription via the canonical BMP-SMAD signaling pathway^13^. Hepcidin transcription is suppressed by iron deficiency, partly via Matriptase-2 (encoded by *TMPRSS6)* mediated downregulation of Hemojuvelin, a coreceptor for BMP signaling. Increased erythropoiesis also suppresses hepcidin via the erythroid-secreted hormone erythroferrone (ERFE)^14^, which acts to inhibit BMP signaling^15^. Hepcidin is also upregulated by inflammation^16^ (via IL6-driven JAK-STAT signaling^17^).

Systemic iron metabolism and PV may be closely intertwined.^18^ Most PV patients present with iron deficiency at diagnosis.^19^ Overt iron deficiency may mask the elevated haemoglobin associated with PV.^20^ Venesection induces iron deficiency to limit further erythropoiesis. Recent reports have indicated that treatment of PV patients with hepcidin analogues^21^ or pharmaceutical upregulation of hepcidin in pre-clinical models of PV^22,23^ may ameliorate disease phenotype. Given emerging interest in the manipulation of iron homeostasis in PV, comprehensive characterization of hepcidin regulation and its role in PV disease is imperative.

Here, we establish and validate an association between disordered systemic iron homeostasis and risk of PV diagnosis through unbiased genome-wide association analyses and evaluate the iron phenotype in a large cohort of PV patients. We then show that hepcidin levels are critical to governing the severity of the erythroid phenotype using preclinical models of Jak2-V617F-driven PV. Further, we demonstrate that in PV, hepcidin is not downregulated by erythroferrone. We also demonstrate an inflammatory phenotype in PV that may influence hepcidin through GP130-coupled receptors. These findings provide novel insights into understanding the pathophysiology of PV and have important implications for new therapeutic interventions.

## Methods

Full details are available in the Supplemental Materials.

### Ethics

Patient samples were collected under Walter and Eliza Hall Institute (WEHI) ethics 18/10LR; or were derived from the Cambridge Blood and Stem Cell Biobank (CBSB) under ethics approval 18/EE/0199 (East of England - Cambridge East Research Ethics Committee). All participants provided written informed consent. The UK Biobank has approval from the UK North West Multi-centre Research Ethics Committee (MREC) as a Research Tissue Bank (approval 21/NW/0157). Use of mice was in accordance with requirements set out by WEHI Animal Ethics Committee (approvals 2017.031 and 2020.034).

### UK Biobank GWAS

We undertook a genome-wide association study (GWAS) of PV cases versus controls. Associations with single nucleotide polymorphisms (SNPs) and small indels were tested genome-wide, using Regenie^24^, under an additive genetic model. Associations included adjustment for sex, age, genotyping array, 10 ancestry principal components, and relatedness. GWAS results were filtered to include only variants with a minor allele frequency (MAF) ≥ 1.2%. For variants within the *HFE* locus, associations with PV were also tested assuming a recessive model, with covariate adjustment as above. Associations at this locus and the four blood cell traits were tested separately in PV cases and controls, using Regenie, as above, under both the additive and recessive models.

### FinnGen GWAS analysis

We utilized the FinnGen resource, data release 6^25,26^. PV cases were identified as having a relevant ICD-8, 9, or 10 code in the hospital discharge register, cause of death register, or cancer register; controls were non-PV individuals without a record of cancer. Genome-wide associations with PV were carried out with adjustment for sex, age, 10 ancestry PCs, and genotyping batch, and assuming an additive genetic model. The difference in the proportion of homozygous AA genotype individuals in PV cases versus controls was tested using Fisher’s Exact Test.

### Patient samples

We analyzed blood from PV patients who fulfilled the World Health Organisation criteria for PV at time of diagnosis (N=30) whose treatment included a history of therapeutic venesection (N=16) or not (N=14); and from healthy controls (N=30). Demographics are presented in Supplemental Table 1.

### Animals

Erythroferrone knockout (*Erfe*-KO)^14,15,27^ and inducible hepcidin knockout (iHamp-KO)^28^ mice have been described previously. Transgenic mice with a single copy Cre recombinase-dependent *Jak2*-V617F transgene located downstream of the *Col1a1* locus (LSL-Jak2-V617F; CreERT2^T/+^) were generated (full methodology in Supplemental Materials). Age- and sex-matched control animals were used in all experiments.

### Bone marrow transplant model of Polycythemia Vera

LSL-Jak2-V617F; CreERT2^T/+^ (PV) or LSL-Jak2-V617F lacking CreERT2 (control) bone marrow (BM) cells were intravenously injected into lethally irradiated Ly5.1/J (B6.SJL-Ptprca Pepcb/BoyJ) recipient mice. Seven weeks post BM transplantation, mice were given tamoxifen (Sigma; 4.2mg in 90% corn oil/10% ethanol) by oral gavage on two consecutive days to induce expression of the mutant *Jak2* allele. Full details in Supplemental Materials.

### Administration of drugs, antibodies, and siRNA

*TMPRSS6* siRNA (Silence Therapeutics GmbH, Berlin, Germany) comprised a double-stranded 19mer RNA oligonucleotide targeting human *TMPRSS6*, linked to a GalNAc unit at the 5’ end of the sense strand enabling hepatic targeting^29^. Non targeting control (NTC) siRNA complimentary to luciferase RNA was used as control. *TMPRSS6* and NTC siRNA were diluted in sterile PBS and 5mg/kg administered by subcutaneous injection every 3 weeks for a total of 3 doses.

Anti-mouse IL6 (clone MPF-20F3) or Rat IgG1 Kappa control antibodies (both made in-house) were administered by intraperitoneal injection every 3 days for a total of 7 doses (500μg per injection) or daily for 5 consecutive days (200μg per injection).

### Statistical analysis

Sample sizes and statistical tests for each experiment are denoted in the figure legends. Data represents mean ± standard deviation. Statistical testing was performed in Prism 9.3.1; GraphPad Software.

For original data contact the corresponding authors.

## Results

### Genome-wide association study links PV diagnosis with *HFE* variants

**Figure 1:**
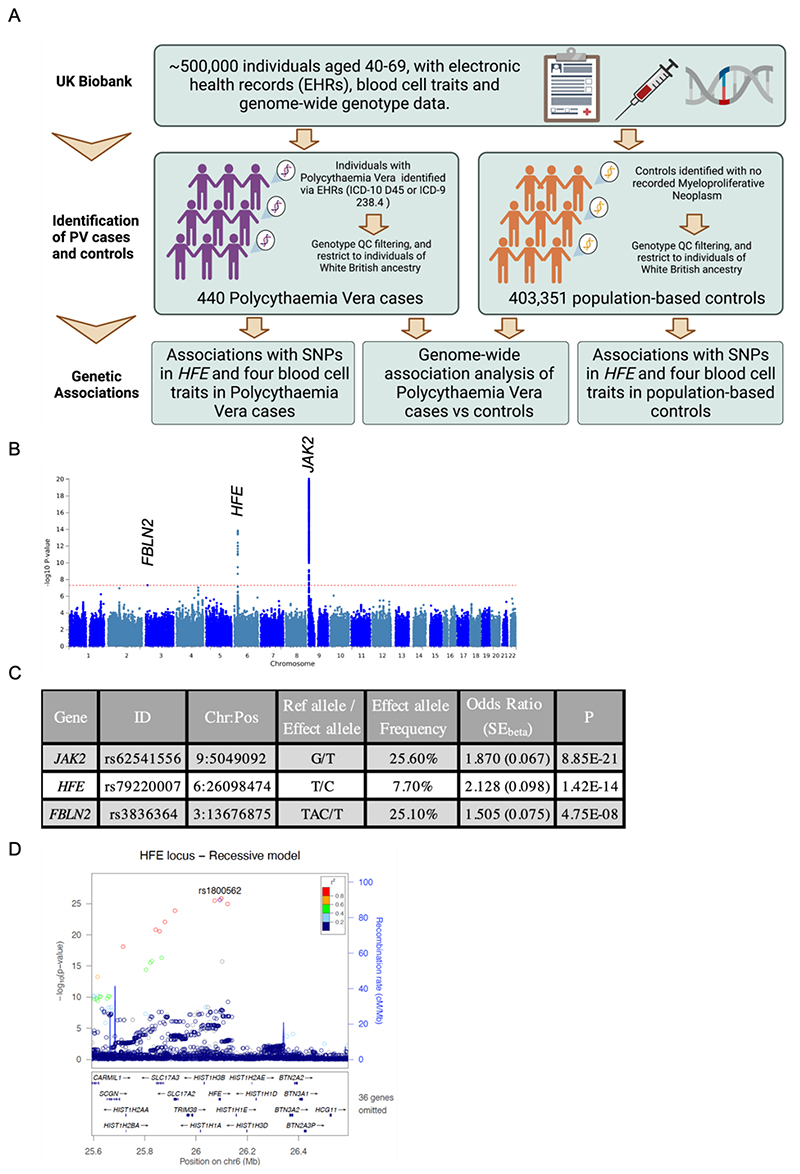
Genome-wide association study (GWAS) of Polycythemia Vera. (A) Schematic of GWAS. Created with BioRender.com. (B) Manhattan plot showing results of GWAS of 440 PV cases vs 403,351 controls, assuming an additive genetic model. Red dotted line shows genome-wide significance level (p<5E-8). Three loci with associations exceeding this threshold are labelled with the nearest gene. (C) Top single nucleotide polymorphism (SNP) at each genetic loci that reached genome-wide significance. (D) LocusZoom plot of associations at the *HFE* locus, assuming a recessive genetic model. rs1800562 (C282Y) highlighted, with the other single nucleotide polymorphisms (SNPs) coloured according to linkage disequilibrium (r^2^) to that SNP.

We undertook a GWAS of 440 PV cases and 403,351 controls in the UK Biobank (fig. 1A). We tested 9,191,064 variants genome-wide (fig. 1B). Three genetic loci had SNPs with genome-wide significant (p<5E-8) associations. Figure 1C details the top SNP at each locus. The most significant association was with rs62541556 (p=8.85E-21), a germline SNP in *JAK2* that tags the 46/1 haplotype implicated in MPNs and is associated with increased susceptibility to V617F-mutant clonal hematopoiesis^30,31^. The other genome-wide significant associations were with rs79220007 in *HFE* (p=1.42E-14) and rs3836364 in *FBLN2* (p=4.75E-8).

Interestingly, the top SNP in *HFE*, rs79220007, is in very high linkage disequilibrium (r^2^=1.0) with rs1800562, a SNP known to cause C282Y, the most common mutation causing *HFE-*related Hemochromatosis, an autosomal recessive disorder producing iron overload^32,33^. Among PV cases, there was an excess of individuals with the AA homozygous (C282Y) genotype, compared to what would be expected under Hardy-Weinberg equilibrium (2.7 expected vs 35 observed). Given the over-representation of AA genotypes in PV cases, and the established role of homozygous C282Y variants in disease, we re-examined the *HFE* locus for associations with PV under a recessive model. The association with rs1800562 was more statistically significant under the recessive model than the additive model (p=2.63E-26 vs p=2.91E-14; Supplemental Table 2, fig. 1D), with the homozygous AA genotype associated with 12.58 times increased odds of PV, compared to G/G or G/A genotypes. Commensurately, there was more than ten-fold increase in the number of individuals with a PV diagnosis for C282Y (AA) homozygous individuals (N = 35, 1.35%, Supplemental Table 3), compared to those with GA or GG genotypes (N=69, 0.12%, and N=336, 0.1%, respectively).

We next sought confirmation of the association of rs1800562 with PV through a look-up of rs1800562 in a GWAS of PV in the independent FinnGen study (394 PV cases vs 217,902 controls)^25,26^. Again, there was an over-representation of the AA genotype amongst PV cases (0.6 expected vs 4 observed). The crude odds ratio comparing the frequency of the AA homozygous genotype in PV cases vs controls in FinnGen was 5.19 (p=8.29E-3, Supplemental Table 4). The FinnGen GWAS assumed an additive genetic model and did not demonstrate an association between rs1800562 and PV (p=0.68). However, the C282Y variant is less frequent in Finnish compared to British populations (MAF 3.7% vs 7.8%), therefore, there may have been limited power to capture the recessive association where an additive effect is assumed.

Using the UK Biobank, we then examined associations between rs1800562 and blood cell traits in PV cases and control individuals separately under both additive and recessive models. In line with a previous GWAS^34^, in control individuals the A allele of rs1800562 was highly associated with higher hemoglobin concentrations, hematocrits, and mean corpuscular volume, but lower erythrocyte count (Supplemental Table 5); this latter association was larger and more statistically significant under a recessive model (Supplemental Table 5).

HFE regulates iron homeostasis by influencing transcription of hepcidin (reviewed^35^). *HFE* knockout mice exhibit marked reductions in liver *Hamp1* mRNA expression.^36^ The C282Y *HFE* variant dysregulates the iron-hepcidin axis, causing lower hepcidin concentrations relative to iron stores^37^. We therefore sought to further investigate how iron homeostasis may affect PV disease.

### Patients with PV exhibit iron deficiency with appropriate reductions in hepcidin expression

**Figure 2:**
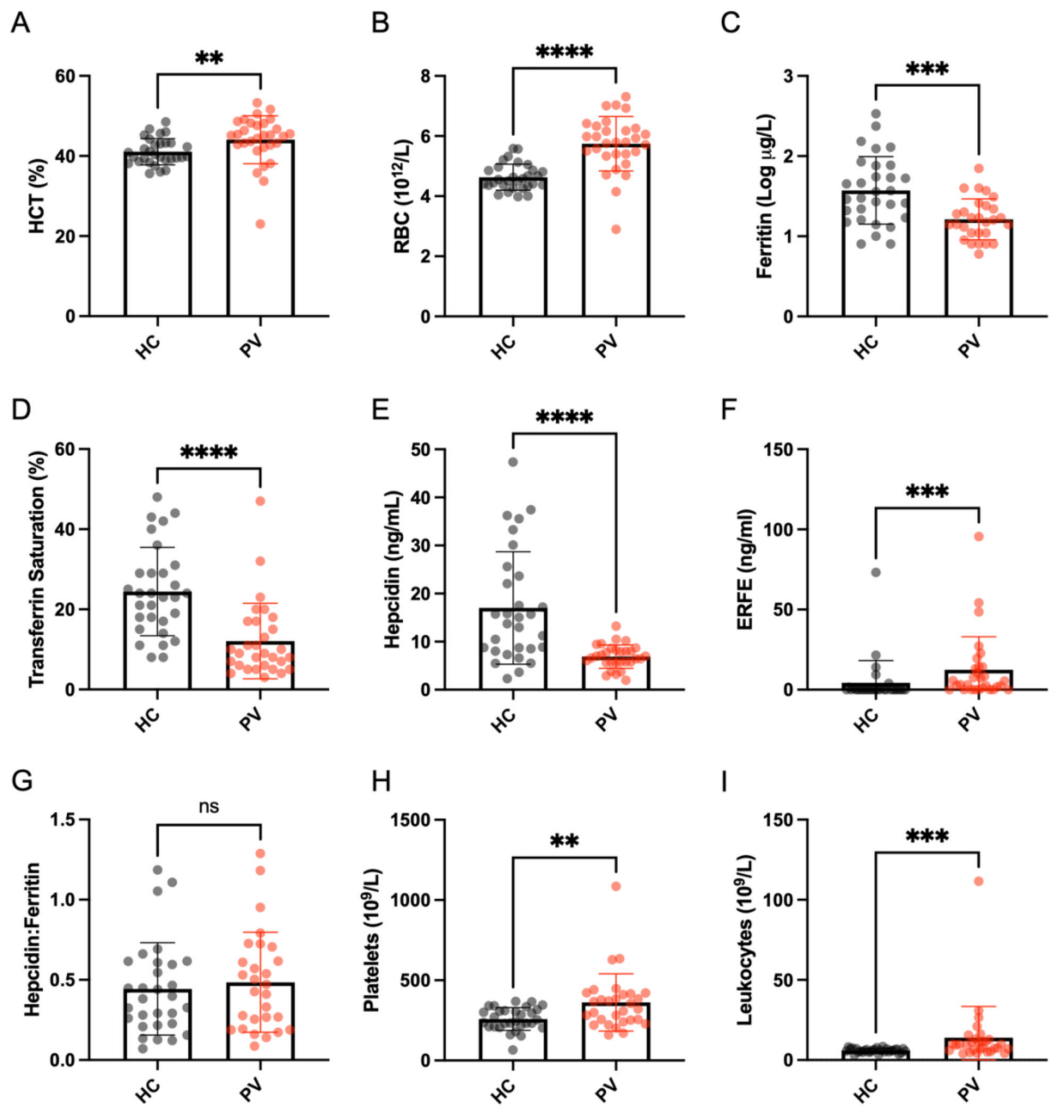
Iron and erythroid parameters in Polycythemia Vera patients and healthy controls. Hematocrit (HCT; A), Red blood cells (RBC; B), Ferritin (C), Transferrin Saturation (D), serum hepcidin (E), serum ERFE, (F) Hepcidin:ferritin ratio, platelet count (H) and leukocyte count (I) of Healthy controls (HC; black) and Polycythemia Vera (PV; red) patients (N=30 PV and HC). Mann-Whitney test (A, D-I) or Unpaired 2-tailed t-test with Welch’s correction (B, C). ** p<0.01; ***p<0.001; ****p<0.0001; ns = non-significant.

We compared haematology and iron markers in 30 patients with PV to 30 healthy controls (Figure 2). PV patients exhibited higher hematocrit and red cell count, and reduced ferritin (log ferritin: PV 1.211 vs control 1.572, p=0.0002) and transferrin saturation (PV 12.1% vs control 24.43%, p<0.0001); hepcidin levels were reduced (PV 6.89 ng/mL vs control 17.01 ng/mL, p<0.0001) and erythroferrone levels increased (PV 12.41 ng/mL vs control 4.26 ng/mL, p=0.0004). The ratio of hepcidin to ferritin was similar between groups (PV 0.485 vs control 0.443, p=0.6048). As expected, PV patients had raised platelet and leukocyte counts. Interestingly, we did not observe significant differences between patients whose treatment included a history of therapeutic venesection and those whose did not (Supplemental Figure 1).

### Hepcidin regulation in a murine inducible knock-in bone-marrow transplant model of Polycythemia Vera

**Figure 3:**
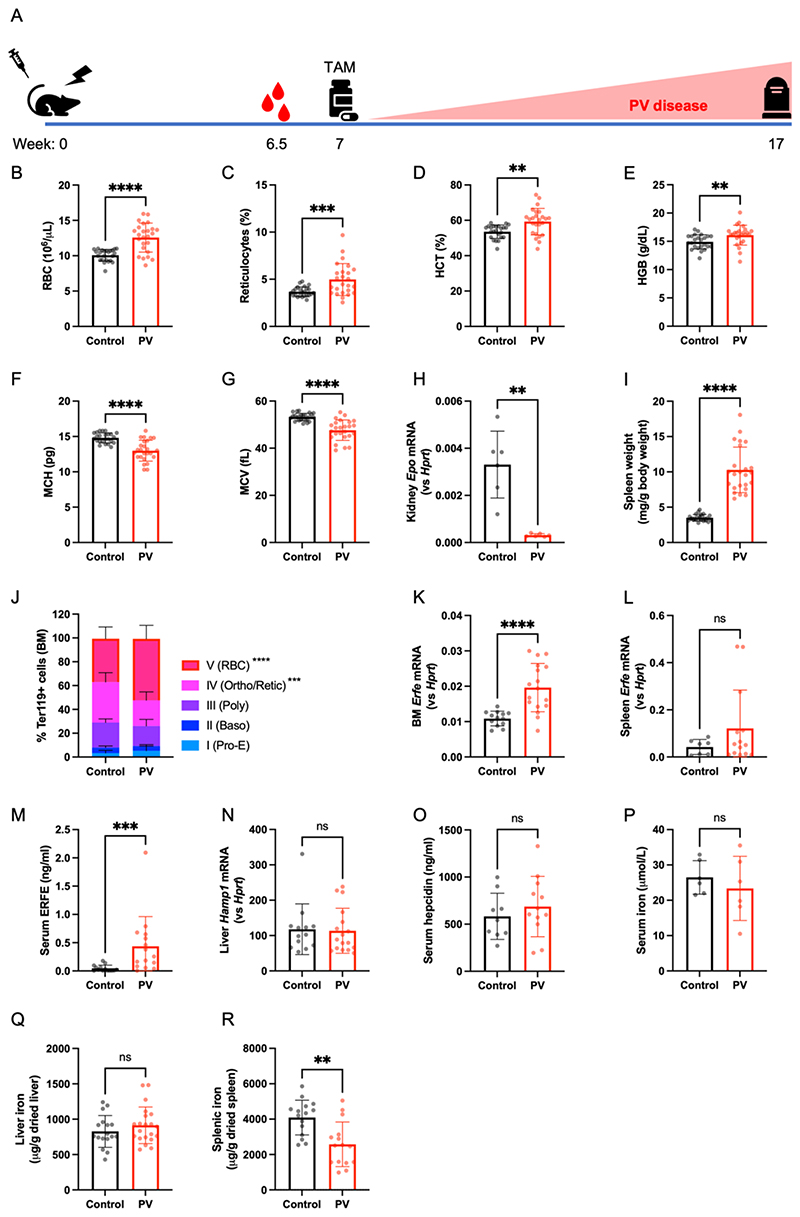
Novel bone marrow transplant mouse model of Polycythemia Vera (A) Schematic of BMT PV mouse model. (B-G) Red blood cells (RBC; B), reticulocytes (C), hematocrit (HCT; D), hemoglobin (HGB; E), mean corpuscular hemoglobin (MCH; F) and mean cell volume (MCV; G) N=24 control/25 PV. (H) Kidney *Epo* mRNA expression relative to *Hprt*. N=6. (I) Spleen weight normalised to total body weight. N=24. (J) Terminal erythropoiesis in the bone marrow determined by flow cytometry. Based on CD44 expression and FSC-A, Ter119+ve cells were gated into 5 distinct populations: I – proerythroblast (Pro- E), II – basophilic erythroblasts (Baso), III – polychromatic erythroblasts (Poly), IV – orthochromatic erythroblasts and reticulocytes (Ortho/Retic), and V – red blood cells (RBC). N=24 control/25 PV. (K) Bone marrow (BM; N=13 control / 17 PV) and (L) spleen (N= 7 control / 14 PV) *Erfe* mRNA expression relative to *Hprt*. (M) Serum ERFE. N=14 control/15 PV. (N) Liver *Hamp1* mRNA expression relative to *Hprt*. N=13 control/17 PV. (O) Serum hepcidin. N=9 control/12 PV. (P) Serum iron. N=6. (Q) Liver (N=17 control/21 PV) and (R) spleen (N=15) non-heme iron content. Mann-Whitney test (B, D, I, L, M, N), Unpaired 2-tailed t-test with Welch’s correction (C, E-H, K, O-R) or two-way ANOVA with Šídák’s correction for multiple comparisons (J). ** p<0.01; ***p<0.001; ****p<0.0001; ns = non-significant.

To define hepcidin regulation in PV we developed a BM transplant model of PV (fig. 3A). Importantly, this model allows inducible mutant *Jak2*-V617F expression in the hematopoietic lineage whilst preserving wildtype JAK2-STAT signaling in hepatocytes. Reconstitution of the BM by donor cells was highly efficient (Supplemental Figure 2). Ten weeks after tamoxifen induction, recipient mice transplanted with LSL-Jak2-V617F; CreERT2^T/+^ BM (hereafter, PV mice) exhibited a classic PV phenotype with elevated red cell count, reticulocytes, hematocrit and hemoglobin, decreased mean corpuscular haemoglobin (MCH) and mean cell volume (MCV), suppressed renal erythropoietin (*Epo*) expression, and splenomegaly (fig. 3B-I); the severity of the phenotype was not inconsistent with human disease. Unlike other murine models of PV^38,39^, PV mice did not have thrombocytosis and exhibited only a ~2-fold increase in leukocytes (Supplemental Figure 3). Compared to controls, PV mice had a skewed distribution of BM resident erythropoietic progenitor cells, with increased levels of the most mature (stage V) erythroid cells (fig. 3J; Supplemental Figure 4). However, in the spleen erythropoiesis was skewed towards increased intermediate progenitors and reduced stage V cells (Supplemental Figure 4). In this PV model, despite increased erythropoiesis and higher *Erfe* mRNA in the BM (but not the spleen) and serum ERFE protein levels (fig. 3K-M), hepcidin mRNA (gene: *Hamp1*) and serum protein levels were not reduced (fig. 3N-O). Serum and liver iron concentrations were similar between PV mice and controls (fig. 3P-Q) but spleen iron was reduced (fig. 3R).

### ERFE does not regulate hepcidin expression in PV

**Figure 4:**
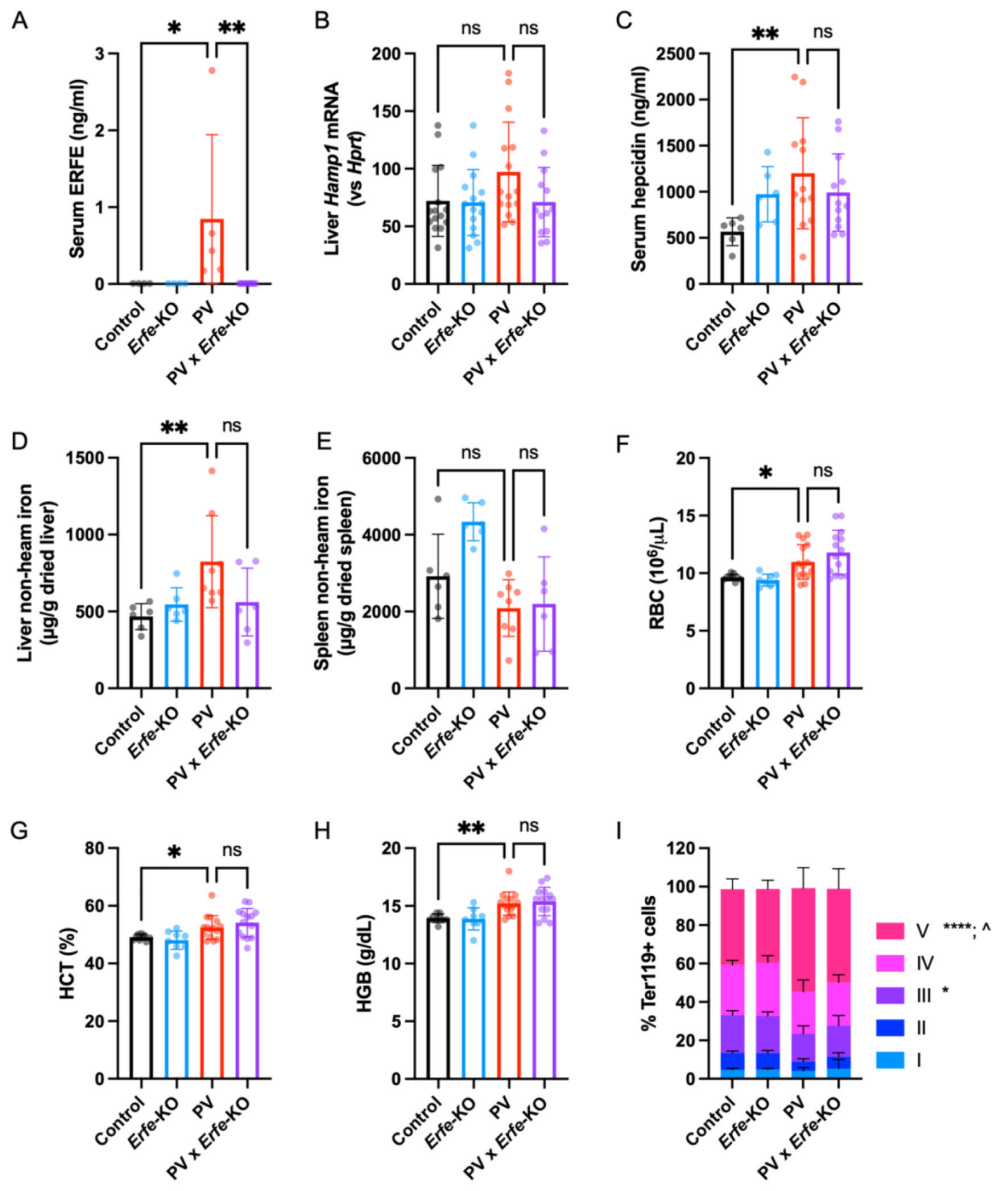
ERFE does not affect hepcidin in Polycythemia Vera (A) Serum ERFE. N=4 control/4 *Erfe-KO/5* PV/11 PV x *Erfe-KO*. (B) Liver *Hamp1* mRNA expression relative to *Hprt*. N=15 control, *Erfe-KO* and PV/13 PV x *Erfe-KO*. (C) Serum hepcidin. N=4 control and *Erfe*-KO/12 PV and PV x *Erfe*-KO. (D) Liver and (E) spleen non-heme iron content. N=6 control, *Erfe*-KO and PV x *Erfe*-KO/8 PV. (F-H) Red blood cells (RBC; F), hematocrit (HCT; G) and hemoglobin (HGB; H). N=9 control/8 *Erfe*-KO/15 PV/14 PV x *Erfe*-KO. (I) Terminal erythropoiesis in the bone marrow determined by flow cytometry. Based on CD44 expression and FSC-A, Ter119+ve cells were gated into 5 distinct populations: I – proerythroblast (Pro-E), II – basophilic erythroblasts (Baso), III – polychromatic erythroblasts (Poly), IV – orthochromatic erythroblasts and reticulocytes (Ortho/Retic), and V – red blood cells (RBC). N=9 control/8 *Erfe*-KO/13 PV/12 PV x *Erfe*-KO. One-way ANOVA (A, C, E-H), Kruskal-Wallis test (B, D) OR Two-way ANOVA with Dunnett’s correction for multiple comparisons (I). * p<0.05; ** p<0.01; ns = non-significant. In (I) * represents Control vs PV and ^ represents PV vs PV x *Erfe*-KO

ERFE levels were modestly increased in our PV model (fig. 3M), a magnitude of increase not dissimilar to the clinical cohort (fig. 2F). To determine the role of ERFE on hepcidin levels and disease severity in PV we crossbred the LSL-Jak2-V617F; CreERT2^T/+^ mice with a previously published *Erfe* knockout (*Erfe*-KO) mouse^15,27,40^ (PV x *Erfe*-KO mice). The inter-crossed mice were used as donors for BM transplants allowing *Jak2*-V617F expression along with *Erfe*-deletion in hematopoietic cells of recipient mice. While PV mice have increased ERFE (fig. 3M), ERFE is undetectable in PV x *Erfe*-KO mice (fig. 4A), however deletion of *Erfe* in PV mice did not alter hepatic *Hamp1* expression (fig. 4B) or hepcidin protein (fig. 4C) or alter systemic iron levels (fig. 4D-E), erythroid parameters (red cell count, hematocrit or hemoglobin; fig. 4F-H), terminal erythropoiesis in the BM (fig. 4I), splenomegaly or other non-erythroid haematological lineages (Supplemental Figure 5).

### Hepcidin determines PV erythroid disease severity

**Figure 5:**
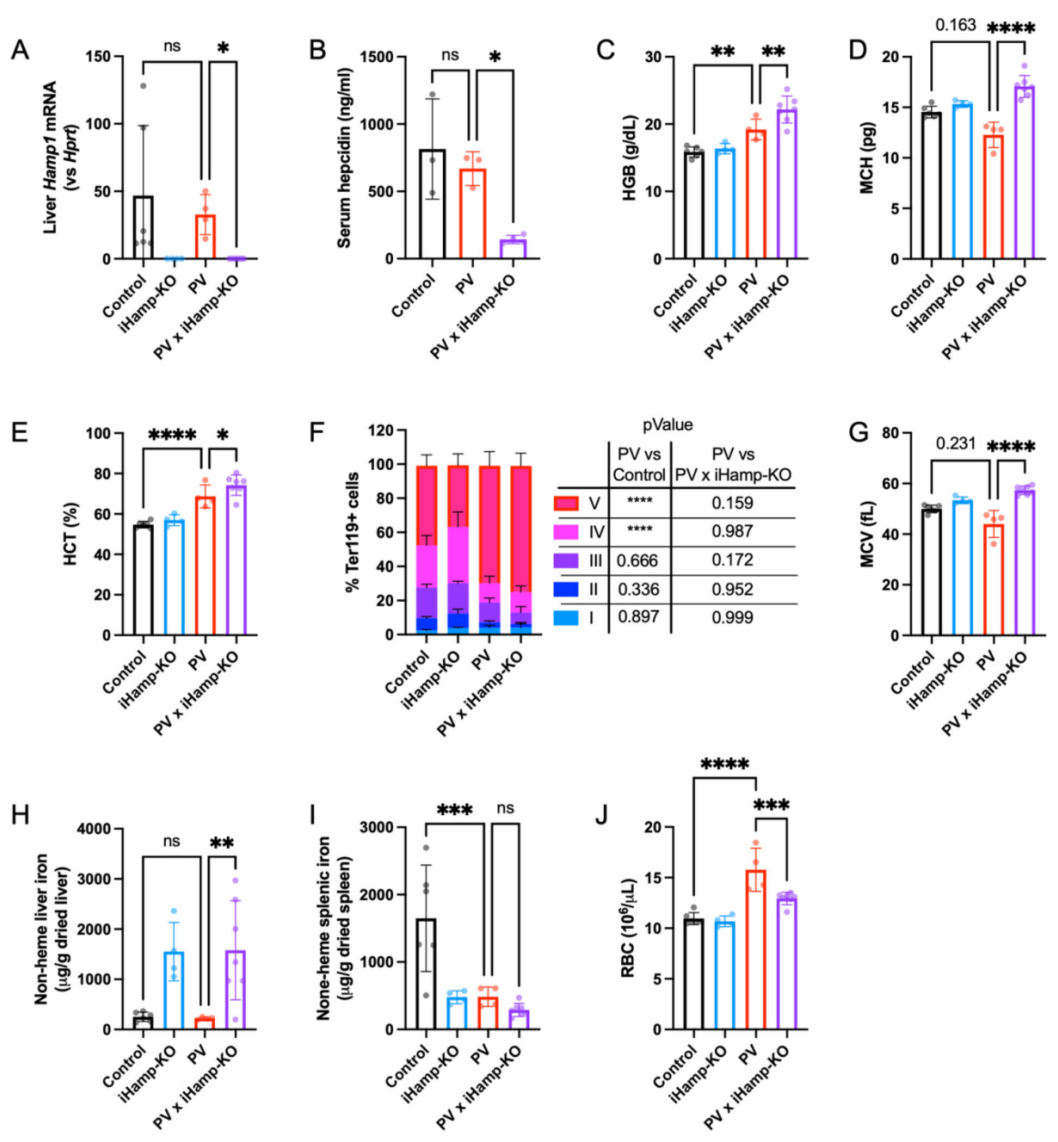
Hepcidin deletion worsens PV erythroid disease severity (A) Liver *Hamp1* mRNA expression relative to *Hprt*. (B) Serum hepcidin. (C – E) Hemoglobin (HGB; C), mean corpuscular hemoglobin (MCH; D) and hematocrit (HCT; E). (F) Terminal erythropoiesis in the bone marrow determined by flow cytometry. Based on CD44 expression and FSC-A, Ter119+ve cells were gated into 5 distinct populations: I – proerythroblasts, II – basophilic erythroblasts, III – polychromatic erythroblasts, IV – orthochromatic erythroblasts and reticulocytes, and V – red blood cells. (G) Mean cell volume (MCV). (H) Liver and (I) spleen non-heme liver iron. (J) Red blood cells (RBC). N=6 Control/4 iHamp-KO/4 PV/7 PV x iHamp-KO. N= 6 Control/4 iHamp-KO/4 PV/7 PV x iHamp-KO except (B) where N = 3 Control/3 PV/4 PV x iHamp-KO. Kruskal-Wallis test (A, D, G), Ordinary one-way ANOVA (B-C, E, H-J) or Two-way ANOVA with Dunnett’s correction for multiple comparisons (F). * p<0.05; ** p<0.01; ***p<0.001; ****p<0.0001; ns = non-significant.

We hypothesized that hepcidin regulates erythroid disease in PV. To test this hypothesis, we first deleted hepcidin in PV mice using a previously published inducible hepcidin knockout (iHamp-KO) mouse model^28^. Since mutant *Jak2*-V617F expression in hepatocytes was not a concern in mice unable to express hepcidin, we crossbred LSL-Jak2-V617F; CreERT2^T/+^ mice with iHamp-KO mice, creating mice with simultaneously inducible systemic mutant *Jak2*-V617F expression and hepcidin deletion (PV x iHamp-KO). As expected, PV x iHamp-KO mice showed no hepatic *Hamp1* expression (fig. 5A) and greatly reduced serum hepcidin protein (fig. 5B). Deletion of hepcidin in PV mice worsened the erythroid phenotype, with PV x iHamp-KO animals, compared to PV mice that express hepcidin (PV animals), having increased hemoglobin concentration (19.20 vs 22.70 g/dL, p=0.0021; fig. 5C), MCH (12.28 vs 17.07 pg, p<0.0001; fig 5D) and hematocrit (68.70 vs 75.85%, p=0.0065; fig. 5E), but did not effect non-erythroid lineages (Supplemental Figure 6). Interestingly, hepcidin deletion did not significantly alter the distribution of erythropoietic cells in the BM (fig 5F). However, the worsened erythroid phenotype may reflect increased iron availability for red cell production as evidenced by increased MCV (43.98 vs 57.62 fl, p=0.0012; fig. 5G) and increased liver iron (fig. 5H) but no change in spleen iron content (fig. 5I). PV x iHamp-KO mice had a lower red cell counts than their hepcidin-expressing controls (15.78 vs 13.16x10^6^/μL, p=0.0052; fig 5J), which, interestingly, recapitulates blood cell traits in control individuals with the *HFE* rs1800562 A allele (Supplemental Table 5). Hepcidin deletion did not affect splenomegaly of PV mice (Supplemental Figure 6).

### TMPRSS6 inhibition increases endogenous hepcidin and improves PV disease

**Figure 6:**
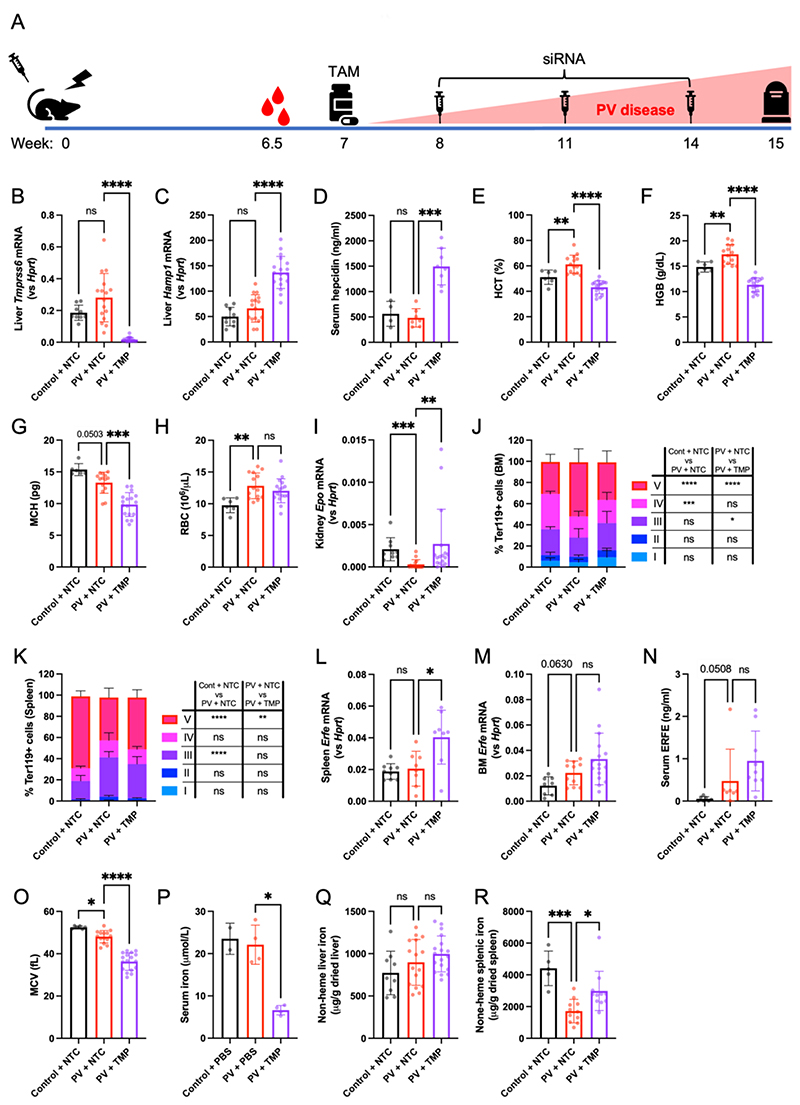
*TMPRSS6* inhibition increases endogenous hepcidin and improves PV disease severity (A) Schematic of experimental design. (B – C) Liver *Tmprss6* (B) and *Hamp1* (C) mRNA expression relative to *Hprt*. N=9 Control + NTC/16 PV + NTC/18 PV + TMP. (D) Serum hepcidin. N=4 Control + NTC/7 PV + NTC/8 PV + TMP. (E-H) Hematocrit (HCT; E), hemoglobin (HGB; F), mean corpuscular hemoglobin (MCH; G) and red blood cells (RBC; H). N=6 Control + NTC/14 PV + NTC/17 PV + TMP. (I) Kidney *Epo* mRNA expression relative to *Hprt*. N=9 Control + NTC/16 PV + NTC/17 PV + TMP. (J-K) Terminal erythropoiesis in the bone marrow (J) and spleen (K) determined by flow cytometry. Based on CD44 expression and FSC-A, Ter119+ve cells were gated into 5 distinct populations: I – proerythroblasts, II – basophilic erythroblasts, III – polychromatic erythroblasts, IV -orthochromatic erythroblasts and reticulocytes, and V – red blood cells. N=7 Control + NTC/13 PV + NTC/19 PV + TMP bone marrow and N=7 Control + NTC/5 PV + NTC/7 PV + TMP spleen. (L-M) Spleen (L) and bone marrow (BM; M) *Erfe* mRNA expression relative to *Hprt*. N=9 Control + NTC/8 PV + NTC/8 PV + TMP spleen and N=8 Control + NTC/11PV + NTC/16 PV + TMP. (N) Serum ERFE. N=7 Control + NTC/7 PV + NTC/8 PV + TMP. (O) Mean cell volume (MCV). N=6 Control + NTC/14 PV + NTC/17 PV + TMP. (P) Serum iron. N=2 control group / 4 PV groups. (Q-R) Liver (Q) and spleen (R) non-heme iron content. N=9 Control + NTC/16 PV + NTC/18 PV + TMP liver and N=5 Control group/11 PV groups spleen. Kruskal-Wallis test (B, D, G, I, L-N, P, R), Ordinary one-way ANOVA (C, E-F, H, O, Q) or Two-way ANOVA with Tukey’s correction for multiple comparisons (J-K). *p<0.05; **p<0.01; ***p<0.001; ****p<0.0001; ns = non-significant.

Since hepcidin ablation worsened the PV erythroid phenotype, we hypothesized that increasing hepcidin levels would impair erythroid iron availability and reduce erythroid disease. Mice were injected with *TMPRSS6* siRNA (that has been shown to cause sustained knockdown of murine *Tmprss6*^29^) or non-targeting control (NTC) siRNA every 3 weeks for a total of 3 injections starting 1-week after tamoxifen induction of the mutant *Jak2*-V617F (fig. 6A), at which time PV mice had a MPN phenotype (Supplemental Figure 7). *TMPRSS6* siRNA treatment resulted in efficient knockdown of hepatic *Tmprss6* (fig. 6B). In PV mice, compared to NTC siRNA treatment, *TMPRSS6* siRNA increased hepatic *Hamp1* 2.07-fold (fig. 6C) and increased hepcidin protein 3.10-fold (fig. 6D).

Hepcidin upregulation by *TMPRSS6* siRNA significantly reduced hematocrit (61.24 vs 43.24%, p<0.0001; fig. 6E), hemoglobin (17.34 vs 11.35 g/dL, p<0.0001; fig. 6F) and MCH (13.29 vs 9.84 pg, p=0.0010; fig. 6G) in PV mice, but did not reduce red cells (12.83 vs 11.81 10^6^/μL, p=0.1458; fig. 6H) and had no effect on non-erythroid lineages (Supplemental Figure 8). *TMPRSS6* siRNA treatment corrected renal *Epo* suppression (fig. 6I) and the distribution of BM (but not splenic) resident erythropoietic progenitor cells in PV mice (fig. 6J-K) but had no effect on splenomegaly (Supplemental Figure 8). *TMPRSS6* siRNA treatment raised *Erfe* mRNA expression in the spleen of PV mice but had no effect on *Erfe* mRNA in the BM or ERFE protein levels in the serum (fig. 6L-N). MCV was reduced in PV mice treated with *TMPRSS6* siRNA (fig. 6O) indicative of iron-restricted erythropoiesis. *TMPRSS6* siRNA significantly reduced serum iron in PV mice (fig. 6P), but did not reduce non-heme liver iron (fig. 6Q) and the treatment raised non-heme splenic iron levels (6R).

### Inflammatory cytokines may upregulate hepcidin in PV

**Figure 7:**
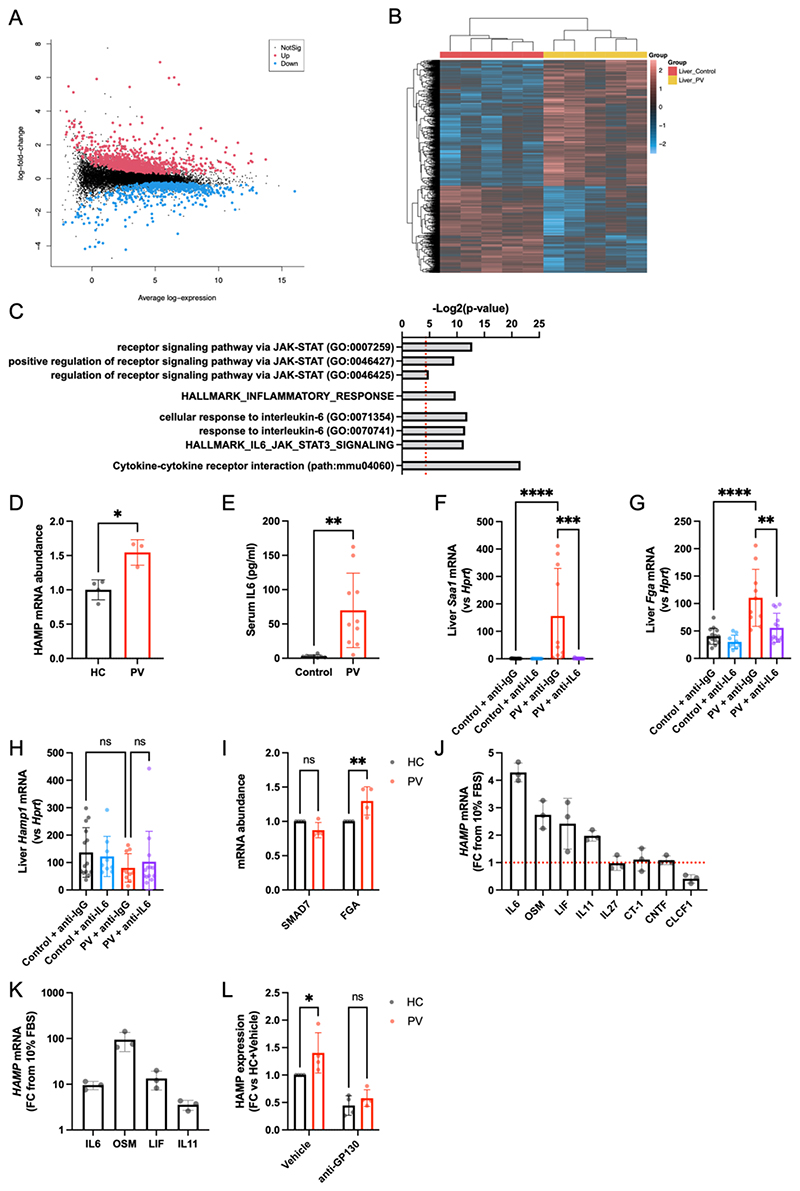
Inflammatory cytokines may upregulate hepcidin in Polycythemia Vera (A) Mean-difference plot showing the average log-expression of each gene (x-axis) and their log-fold change between PV and control liver samples (y-axis). The differentially expressed genes (DEGs) are highlighted with points in red and blue indicating upregulated and downregulated genes, respectively (adjusted p-value <0.05). (B) Heatmap of the expression of all DEGs with hierarchical clustering where expression values are standardised to have mean 0 and standard deviation 1 for each gene. (C) Bar chart depicting Gene Ontology (GO) biological processes, MSigDB hallmark gene sets or KEGG pathways relating to JAK-STAT signalling, inflammatory response, interleukin-6 (IL6) responses or cytokine-cytokine receptor interactions that are associated with upregulated genes in PV liver samples vs control. X-axis represents statistical significance of the enrichment, increasing from left to right. Red dashed line represents p=0.05. (D) *HAMP* mRNA expression of HepG2 cells cultured in media supplemented with 2% plasma from healthy control donors (HC) or PV patients. N=4 HC/3 PV. (E) Mouse serum interleukin-6 (IL6). N=8 Control/10 PV. (F-H) Liver *Saa1* (F), *Fga* (G) and *Hamp1* (H) relative to *Hprt*. N=14 Control + anti-IgG/9 Control + anti-IL6/10 PV + anti- IgG/13 PV + anti-IL6. (I) *SMAD7* and *FGA* mRNA expression of HepG2 cells cultured in media supplemented with 2% plasma from healthy control donors (HC) or PV patients. N=4. (J-K) *HAMP* mRNA expression of HepG2 (J) or Huh7 (K) cells cultured in media supplemented with 10ng/ml recombinant human interleukin-6 family cytokines. N=3. The red line in (J) indicates *HAMP* expression in the absence of additional cytokines. (L) *HAMP* mRNA expression of HepG2 cells cultured in media supplemented with 2% plasma from healthy control donors (HC) or PV patients with the addition of anti-GP130 antibodies or vehicle control (PBS). N=4. Unpaired 2-tailed t-test with Welch’s correction (D, E), Kruskal-Wallis test (F-H) or Two-way ANOVA with Šídák’s correction for multiple comparisons (I, L). *p<0.05; ** p<0.01; ***p<0.001; ****p<0.0001; ns = non-significant. IL6 – Interleukin-6; OSM – Oncostatin M; LIF – Leukemia inhibitory factor, IL-11 – Interleukin-11; IL-27 – Interleukin-27, CT-1 – Cardiotrophin 1; CNTF – Ciliary neurotrophic factor, CLCF1 – Cardiotrophin-like cytokine factor

Inflammatory cytokines (including IL6) are elevated in PV^41,42^. Hepcidin transcription is directly regulated via inflammation, via IL6-mediated JAK-STAT signaling^17^. Thus, we examined whether JAK-STAT signaling was increased in hepatocytes of PV mice. RNA-Seq analysis on livers from BM transplanted PV and control animals revealed 1466 (598 downregulated; 868 upregulated) differentially expressed genes (DEGs; fig. 7A and Supplemental Table 6) in PV mice compared to controls. The transcriptional profile of livers from PV mice was different from controls (fig. 7B). Gene Ontology (GO) pathway analysis of DEGs revealed upregulation of biological processes related to JAK-STAT signaling in PV mice compared to control (fig. 7C, top 3 bars). Consistent with this analysis, gene set enrichment analysis (GSEA) using the MSigDB hallmark gene sets revealed upregulation of the inflammatory response pathway in the liver of PV mice (fig. 7C, fourth bar).

We thus explored the hypothesis that soluble factors (including cytokines) may upregulate hepcidin in PV patients using a human *in vitro* hepatocyte model. Liver-derived HepG2 cells grown in media supplemented with plasma from PV patients exhibited increased hepcidin (*HAMP*) mRNA expression compared to cells grown in media supplemented with healthy donor plasma (fig. 7D), indicating PV patients produce soluble factors that upregulate hepcidin. We reasoned that IL6 may be driving this upregulation since PV mice exhibited increased serum IL6 (fig. 7E). In addition, RNA-seq GO pathway analysis, as well as GSEA using the MSigDB hallmark gene sets, revealed increased IL6-driven responses in the livers of PV mice (fig. 7C, fifth to seventh bars). To determine whether IL6 alone is responsible for hepcidin upregulation in PV, BM-transplanted mice were treated with anti-IL6 or control (anti-IgG) antibodies. Mice treated with anti-IL6 antibodies every 3 days for 3 weeks showed normalization of IL6-JAK-STAT (e.g. *Saa1*) transcripts and reduced expression of hepatic JAK-STAT transcripts (e.g. *Fga*) (fig. 7F-G), confirming neutralization of IL6 signaling. However, IL6 neutralization did not alter hepcidin expression (fig. 7H). These results were replicated in mice treated with anti-IL6 daily for 1 week (Supplemental Figure 9). In keeping with unaltered hepcidin expression, anti-IL6 treatment had a minimal effect on erythropoiesis or iron distribution in PV mice (Supplemental Figure 10).

We therefore hypothesized other cytokines may upregulate hepcidin in PV. In keeping with this, KEGG pathway analysis of DEGs revealed upregulation of genes involved in cytokine-cytokine receptor interaction in the liver of PV mice compared to controls (fig. 7C, bottom bar). HepG2 cells cultured in media supplemented with plasma from PV patients exhibited increased JAK-STAT target gene expression (*FGA*) but no change in BMP-SMAD target gene expression (*SMAD7*) when compared to cells cultured in media supplemented with plasma from healthy donors (fig. 7I). Notably, levels of other IL6-family cytokines (IL11^43,44^, OSM^45^) are known to be elevated in PV. Thus, we screened other IL6-family cytokines for a role in hepcidin regulation since these cytokines induce JAK-STAT signaling via a common GP130 receptor subunit (except IL31). Addition of individual IL6-family cytokines to HepG2 cells revealed that in addition to IL6, IL11, OSM, and LIF increase *HAMP* mRNA levels; whereas IL27, CT-1, and CNTF have no effect and CLCF1 decreases hepcidin expression (fig. 7J). The increase in hepcidin expression by IL11, OSM and LIF (as well as IL6) was confirmed in a second hepatocyte cell line, Huh7 (fig. 7K). We then determined whether inhibition of GP130 could normalize hepcidin expression in HepG2 cells treated with PV plasma. HepG2 cells grown in media supplemented with PV plasma and anti-GP130 antibodies no longer exhibited increased *HAMP* expression, rather *HAMP* expression decreased, reaching levels indistinguishable from that of cells cultured in media supplemented with control plasma and anti-GP130 antibodies (fig. 7L).

## Discussion

Here, we provide population genetic and experimental evidence that diagnosis and clinical features of PV are influenced by systemic iron homeostasis. This work establishes a central role for iron homeostasis in influencing the erythroid phenotype in PV and provides a rationale for the use of therapies that modify iron metabolism to treat this disease.

Our GWAS implicates the *HFE* locus as a key region associated with PV diagnosis, formally linking systemic iron regulation and PV. The top SNP in *HFE* associated with PV is in very high linkage disequilibrium with the pathogenic HFE C282Y variant (which causes hemochromatosis). Interestingly, this disease-causing SNP is not associated with diagnosis of other MPNs (Essential Thrombocythemia and Primary Myelofibrosis, Supplemental Table 7), indicating iron metabolism is linked to PV exclusively. Provision of iron to PV may worsen hematocrit^46^. Conversely, as we show, iron deficiency is common in patients with PV^19^, and may conceal diagnosis^47^. Previous small studies have examined the prevalence and effects of HFE C282Y variants in PV and have not observed associations^48,49^. However, here, utilization of large datasets enabled an unbiased approach to discover and validate the role of HFE in PV diagnosis. Cases of PV were defined via the ICD system and subject to contemporaneous criteria at the time of disease diagnosis.

HFE is an upstream regulator of hepcidin, perhaps via stabilisation of BMP-type I receptors (ALK3) to facilitate BMP signaling.^50,51^ C282Y variants prevent HFE from reaching the cell surface, limiting its function, reducing hepcidin expression, and causing excess iron absorption^37,52^. *HFE* variants may thus enhance iron availability for erythropoiesis, increasing the likelihood of presentation and diagnosis of PV.

Given the established role of HFE in hepcidin regulation, we reasoned the ultimate mechanism for the genetic association with disease phenotype was via changes in hepcidin, and hence we dissected the role of hepcidin in PV using pre-clinical models. Genetic deletion of hepcidin in our PV model increased erythroid parameters, indicating that in PV, unrestricted erythroid iron access results in unfettered increased hematocrit and hemoglobin production. Conversely, physiologic upregulation of hepcidin expression through RNA interference of *Tmprss6* induced reductions in serum iron, depriving the BM of iron and causing reduced hematocrit and hemoglobin concentration. Hepcidin is thus critical in determining the erythroid phenotype in PV.

The dependence of the PV erythroid phenotype on systemic iron homeostasis provides the mechanistic rationale for the suite of emerging treatments that target iron metabolism for PV. The mainstay of current treatment for PV is venesection to decrease hematocrit below 45%, at which serious thrombotic events become less likely^8,53^. Venesection lowers the hematocrit by removing red cells and inducing systemic iron deficiency,^18^ thus ameliorating further erythrocyte production^53^. However, venesection can cause unwanted adverse effects including vasovagal reactions due to fluid shifts^54^, as well as symptoms such as chronic fatigue due to systemic iron deficiency^55^, and incurs direct and indirect health care costs associated with patient visits. Some patients are unable to tolerate venesection due to the severity of adverse events or do not achieve satisfactory hematocrit responses and hence require second-line non-targeted agents. New therapeutic options for PV are thus needed. Withholding iron from the BM by inhibiting iron export to the plasma offers an exciting therapeutic opportunity to replace therapeutic venesection to treat PV.^56^ We demonstrated that upregulation of endogenous hepcidin levels through liver-specific *TMPRSS6* siRNA deprives the serum of iron and reduces hematocrit and hemoglobin concentration in our PV model. A similar approach using anti-sense oligonucleotide therapies has likewise recently shown promising preclinical results^22^. A Phase 2 clinical trial showed that a hepcidin mimetic can obviate the need for venesection in previously venesection-dependent PV patients^21^. A clinical candidate of the *TMPRSS6* siRNA used here (SLN124) is entering Phase 1 and 2 clinical trials (NCT05499013) in PV patients. Our data indicate that liver iron stores were not altered by *TMPRSS6* siRNA treatment, but splenic iron stores increased, consistent with this therapy redistributing iron stores rather than inducing systemic iron depletion, a feature that could protect patients from symptomatic iron deficiency. *TMPRSS6* siRNA treatment is unlikely to result in vasovagal responses. Since hepcidin mimetics and *TMPRSS6* siRNA therapies can be administered by subcutaneous injection, these therapies could potentially be self-administered, reducing healthcare costs.

In our clinical study, we found that patients exhibit low iron stores with concordant reductions in hepcidin that are not likely influenced by small increases in ERFE. Iron depletion and low iron stores have been observed in some previous studies^18,57^ although others have reported hepcidin levels similar to healthy controls.^58,59^ Although we detected elevated ERFE in our PV model and PV patients, *Erfe* deletion did not modify hepcidin expression or alter disease phenotype. These findings may reflect the smaller degree of ERFE elevation in PV compared with diseases of ineffective erythropoiesis such as thalassemia^40^. In keeping with this, we found that hepcidin:ferritin ratios are not reduced in PV patients, unlike in thalassemia where ratios are lower.^60^ Elevations of ERFE in our PV model were comparable to elevations detected in experimental ‘low’ level ERFE-overexpressing mice, which do not alter hepcidin expression and produce only modest elevations in serum and liver iron^61^.

Numerous inflammatory cytokines (including IL6) are elevated in PV patients, some of which may portend inferior prognosis^42,44,62^. We found preliminary evidence to suggest IL-6 family cytokines (beyond IL-6 itself), which signal via GP130-coupled receptors^63^, may be implicated in hepcidin regulation in PV. Consistent with our data, previous studies have indicated that anti-IL-6 treatment of PV mice does not alter hematologic phenotype.^64^ Further characterization of the role of these cytokine(s) in hepcidin regulation in PV will be an important continuation of this work.

Characterization of the functional role of hepcidin and erythroferrone in PV was undertaken in a novel knock-in inducible *Jak2*-V617F transplant model, with induction of the phenotype by tamoxifen delayed to ensure recovery of the mouse from the toxicity of the transplant procedure, which could itself induce inflammation and perturb hepcidin.^65^ The hematologic and splenic phenotype of this model, and the degree of upregulation of erythroferrone, was consistent with human PV disease although less severe than previous knock-in models of *Jak2-*V617F PV which have exhibited a stronger phenotype, with higher hemoglobin and hematocrit levels and greater degrees of splenomegaly;^22,39,66,67^ these models may exhibit more marked elevations in erythroferrone, and deletion of erythroferrone in these models may have induced a change in hepcidin levels and changes in systemic iron physiology. Evaluation of the role of erythroferrone in alternative models of PV remains an ongoing research need.

In summary, our findings implicate systemic iron regulation as a key determinant of the clinical severity of PV and lay the foundation for strategies that modify iron regulation as potential therapeutics for this disease.

## Supplementary Material

Supplementary material

Supplementary tables

## Key Points

Homozygou*s HFE* mutations are over represented in Polycythemia Vera patients

Hepcidin levels govern severity of, and can be manipulated to modify, erythroid phenotype in Polycythemia Vera
