# Supplementary material for: Iron homeostasis governs erythroid phenotype in Polycythemia Vera
Source: Blood. Author manuscript; Available in PMC 2024 Feb 15. (PMC10646816; doi:10.1182/blood.2022016779)
Supplement: Supplementary material [file EMS188510-supplement-Supplementary_material.pdf]

Supplemental Data

Supplemental Methods

UK Biobank – defining Polycythemia Vera cases, and blood cell trait phenotypes

Data from British individuals in the UK Biobank dataset[1] were analysed. PV cases were identified using Hospital Episode Statistics (HES). Individuals were classified as a case if they had a relevant ICD code (ICD-10 of D45 or an ICD-9 of 238.4) recorded under any of the following: Underlying (primary) cause of death; contributory (secondary) causes of death; Diagnoses (main); Diagnoses (secondary). Control individuals who did not have an ICD-9 or -10 code relating to any Myeloproliferative Neoplasm (PV - as above, Essential Thrombocythemia ICD-10, D47.3/ ICD-9, 238.71 or Primary Myelofibrosis ICD-10, D47.4 / ICD-9, 289.83). Individuals without HES data available were excluded.

Cleaning of four blood cell traits (hemoglobin concentration, hematocrit percentage, mean corpuscular volume, and erythrocyte count) was undertaken as previously reported[2]. Briefly, all traits were first log-transformed, or logit transformed (hematocrit percentage). Using a restricted set of central measurements (measures <3.5 median absolute deviations from the median), a generalised additive model (GAM, using the mgcv R package) was fitted, to model the effects of several technical covariates (time of measurement, instrument, acquisition route, day of the week). In the full dataset, model residuals were calculated and used to generate traits adjusted for technical effects. Finally, outliers (>6 median absolute deviations from the median) were excluded. The log (or logit) transformed, adjusted traits were used for downstream genetic analyses.

UK Biobank – genetic data quality control and genome-wide association analysis

The genotyping procedure, quality control and imputation of the UK Biobank cohort is described in detail elsewhere[1]. Individuals with outlying heterozygosity or high levels of missingness were excluded prior to imputation. We further excluded individuals who had withdrawn consent, samples where the self-reported sex did not match the genetically inferred sex, samples with putative sex chromosome aneuploidy, and samples with an excess of relatives (>10 3rd degree). Samples were then restricted to the "White British" subset[1]. Variants were filtered to include those with MAF>0.01%, and INFO>0.8.

Source code can be accessed at: https://github.com/bahlolab/polycythemiaVeraGWAS

Generation of Cre recombinase-inducible Jak2-V617F transgenic mice.

Complementary DNA (cDNA) encoding murine *Jak2*-V617F was cloned downstream of a CMV enhancer-chicken beta-actin promoter element with an intervening cassette including 3 transcriptional termination elements flanked by loxP sites (modification of plasmid pCAGGS-loxSTOPlox-ClaI flp-in, obtained from Addgene; deposited by Rudolf Jaenisch). This was then inserted downstream of the *Col1a1* locus by FRT/Flpe recombinase-mediated site-specific integration by co-transfection with a Flpe expression vector into C57BL/6 5B3 ES cells containing an FRT-hygro-pA "homing" cassette at the ColA1 gene. Correctly targeted ES cell clones were injected into Balb/c blastocysts to generate chimeric mice. Male chimeras were mated with C57BL/6 females to yield heterozygotes for the targeted allele, referred to as LSL-Jak2-V617F mice. To enable tamoxifen-dependent activation of Jak2-V617F expression, LSL-Jak2-V617F mice were intercrossed with previously described CreERT2 mice[3].

Bone marrow transplant model of Polycythemia Vera

Bone marrow cells were flushed from the tibias and femurs of LSL-Jak2-V617F; CreERT2$^{T/+}$ mice (or LSL-Jak2-V617F lacking CreERT2 for controls) with 5% FBS (Gibco) in KDS BSS (150mM Sodium Chloride, 3.7mM Potassium Chloride, 2.5mM Calcium Chloride Dihydrate, 1.2mM Magnesium Sulfate Heptahydrate, 14.8mM HEPES) and passed through a 23G needle to produce a single cell solution under sterile conditions. Bone marrow cells were then pelleted under centrifugation (470g, 5min) and resuspended in 2% FBS/KDS BSS. 2.5x10^6 donor bone marrow cells were intravenously injected into lethally irradiated (two 5.5 Gy doses given at least 4 hours apart) Ly5.1/J (B6.SJL-Ptprca Pepcb/BoyJ) recipient mice. Irradiated mice were given antibiotics (1.1mg/ml neomycin) in their drinking water for 3 weeks post irradiation. Seven weeks post bone marrow transplantation, mice were given tamoxifen (Sigma; 4.2mg in 90% corn oil/10% ethanol) by oral gavage on 2 consecutive days to induce expression of the mutant *Jak2* allele (Supplemental Figure 11). Mice were then humanely euthanised 8-10 weeks later.

Chimerism assessment

Six and a half weeks after bone marrow transplantation, mice were bled from the retro-orbital plexus into Microvette® 500 K3 EDTA tubes (Sarstedt). 100µl blood was added to 10ml red cell lysis buffer (156mM Ammonium Chloride, 11.9mM Sodium Bicarbonate, 0.097mM EDTA) and then pelleted under centrifugation (470g, 5min). The cell pellet was washed once in 4.5ml FACS buffer (2% FBS, 0.002% Sodium Azide in KDB BSS), then resuspended in FACS buffer containing CD45.1-PE (clone A20, made in house) and CD45.2-A647 (clone S450-15.2, made in house) and incubated on ice for 30 mins. Cells were washed once in FACS buffer and resuspended in 100µl FACS buffer and 50uL Hydroxystilbamidine (25µg/mL; Biotium). Samples were run on a BD FACSymphony A3 using BD Diva software. Samples

were analysed using FlowJo 10.8.0 (BD). Chimerism was defined as the percentage of live cells expressing CD45.2.

Gene expression analysis

Total RNA from liver, spleen, kidney, and bone marrow samples and HepG2 and Huh7 cells were isolated using the ISOLATE II RNA Mini Kit (Bioline). 500ng RNA was reverse-transcribed to cDNA using the SensiFAST cDNA synthesis kit (Bioline), according to the manufacturer's instructions. Gene expression levels were measured by RT-qPCR using SensiFAST SYBR No-ROX kit or SensiFAST Probe No-ROX kit (both Bioline) on a LightCycler® 480 II (Roche). Primers or probes used for RT-qPCR are found in Supplemental Table 8. All probes were purchased from ThermoFisher Scientific.

Analysis of bone marrow erythropoiesis by FACS

Bone marrow cells were flushed from the tibias and femurs and a single cell solution created as above. $2x10^6$ cells were pelleted under centrifugation (470g, 5min, 4ºC), resuspended in 50µl FACS buffer containing anti-mouse CD44-FITC (clone: IM781, made in house) and anti-mouse TER119-APC (clone TER119, made in house) and incubated on ice in the dark for 30 minutes. Cells were washed in 4.5ml FACS buffer, pelleted under centrifugation (470g, 5min, 4ºC) and resuspended in 100µL FACS buffer. 50µL Hydroxystilbamidine (25µg/mL – Biotium) was then added before running samples on a BD FACSymphony cytometer using BD Diva software. Sample analysis was performed using FlowJo 10.8.0 (BD). Gating of erythroid cell populations has been previously described[4].

RNA-Sequencing

300ng RNA was used as input for purifying the poly(A)-containing mRNA molecules, RNA amplification, and synthesis of double-stranded cDNAs according to Illumina's TruSeq RNA Sample Prep guidelines. The indexed libraries were pooled and diluted to 1.5pM for paired end sequencing (2x 76 cycles) on a NextSeq 500 instrument using the v2 150 cycle High Output kit (Illumina) as per manufacturer's instructions. The base calling and quality scoring were determined using Real-Time Analysis on board software v2.4.6, while the FASTQ file generation and de-multiplexing utilised bcl2fastq conversion software v2.15.0.4.

Paired-end RNA sequencing reads were aligned to the mm10 build of the mouse reference genome using the Rsubread package v2.4.3[5]. Over 98% of fragments (read pairs) mapped to the reference genome for each sample. Successfully mapped fragments were then summarized into gene-level counts using featureCounts[6] and genes were identified using Gencode annotation to the mm10 genome (version M25)[7] where 83-86% of mapped fragments were assigned to genes. Differential expression analyses were carried out using limma v3.48.3[8,9] and edgeR v3.34.0[10].

After excluding genes that were to be experimentally confirmed, expression-based filtering was carried out using the filterByExpr function with default settings in edgeR. A total of 15,690 genes remained for downstream analysis. Compositional differences between the libraries were normalized using the trimmed mean of M-values (TMM) method[11]. Counts were then transformed to $\log_2$ counts per million (logCPM) with associated observational-level weights using voom[12]. Differential expression between PV and control samples was assessed using linear models and robust empirical Bayes moderated t-statistics. Furthermore, the linear models incorporated a batch effect correction for assay effect to increase precision. False discovery rate was controlled below 5% using the Benjamini and Hochberg method. The mean-difference

plot was generated using the limma's plotMD function while the heatmap was created using the pheatmap software package.

Pathway analyses were performed on differentially expressed genes to test for overrepresentation of biological pathways as defined by Gene Ontology (GO)[13,14] and Kyoto Encyclopedia of Genes and Genomes (KEGG) pathways[15-17] using limma's goana and kegga functions respectively. Analysis of the Molecular Signatures Database (MSigDB) hallmark gene sets[18,19] was undertaken using the fry gene set test in limma.

Analysis of hematological and iron parameters

Full blood counts were determined using an Advia2120i on EDTA anticoagulated blood. Serum iron was measured using an Abbott ARCHITECT analyser using the MULTIGENT Iron assay. Tissue non-heme iron was measured as previously described[20]. Hepcidin (Intrinsic Life Sciences), erythroferrone (Intrinsic Life Sciences) and interleukin-6 (R&D Systems) ELISAs were performed following manufacturer's instructions.

Patient samples

Full blot counts were determined using a Mindray BC-20s or Coulter LH 750 haematology analyser on blood collected into EDTA. Blood was collected in Becton Dickenson (BD) Vacutainer tubes or S-Monovette Z Gel clot activator tubes (Sarstedt), and serum aliquots stored at −80°C or in liquid nitrogen (Cambridge Blood Stem Cell Biobank). Ferritin and transferrin saturation were measured using an Abbott ARCHITECT analyser using the MULTIGENT Iron assay. For cell culture experiments, blood from JAK2 V617F positive patients or healthy controls was collected in Becton Dickenson (BD) Vacutainer EDTA tubes and plasma stored at –80°C.

Cell culture

HepG2 and Huh7 cells were maintained in Dulbecco's modified Eagle medium (DMEM; Gibco) containing 1g/L D-Glucose, 1% L-glutamine, 100mg/L Sodium Pyruvate and supplemented with 1% penicillin-streptomycin and 10% FBS (Gibco). $3x10^5$ cells were seeded in wells of 24-well tissue culture treated plates. After 24h, 10ng/ml recombinant human IL6-family cytokines (IL6, IL11, OSM, LIF, IL27, CT-1, CTNF and CLCF1 all R&D Systems) were added to the media; or media was removed and replaced with media supplemented with 2% human plasma (instead of FBS) with or without the addition of 300ng/ml anti-human GP130 (clone 28105; R&D Systems). In all cases cells were lysed 24 hours later and RNA isolated for RT-qPCR as above.

Supplemental Tables

Supplemental Tables 1 to 8 are supplied as an additional excel file

Supplemental Figures

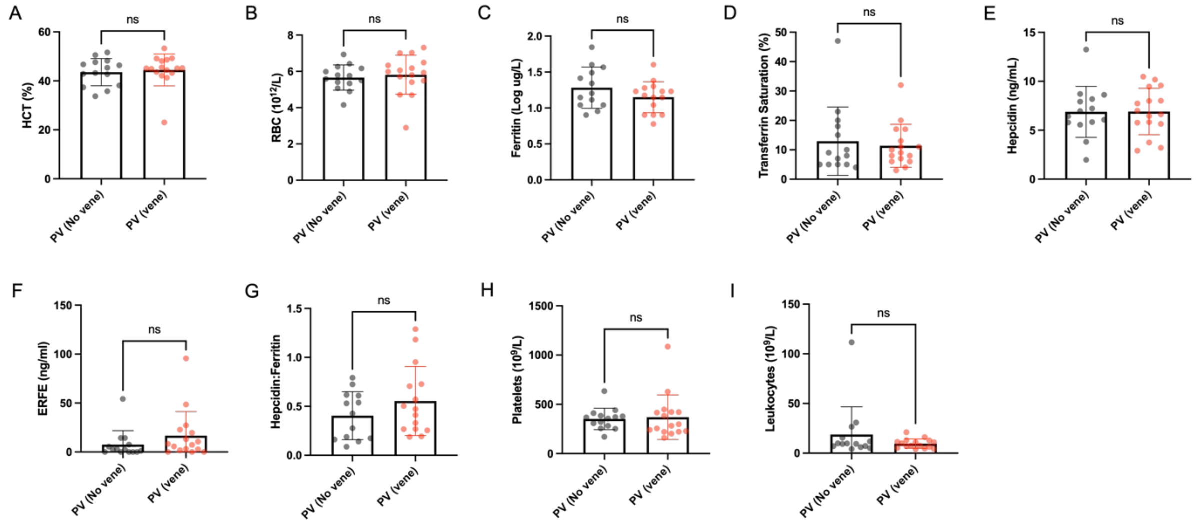

Supplemental Figure 1 – Haematological and iron markers are similar in PV patients with and without history of venesection. (A) Hematocrit (HCT); (B) red blood cell count (RBC); (C) ferritin; (D) transferrin saturation; (E) serum hepcidin; (F) serum ERFE, (G) hepcidin:ferritin ratio, (H) platelet count and (I) leukocyte count in PV patients with a history of venesection (red dots) or those without (black dots). (A, B, D-F, H-I) N=14 no vene/16 vene; (C, G) N=13 no vene/15 vene. Mann-Whitney test (A, D, F, G-I) or Unpaired 2-tailed t-test with Welch's correction (B, C, E). ns = non-significant

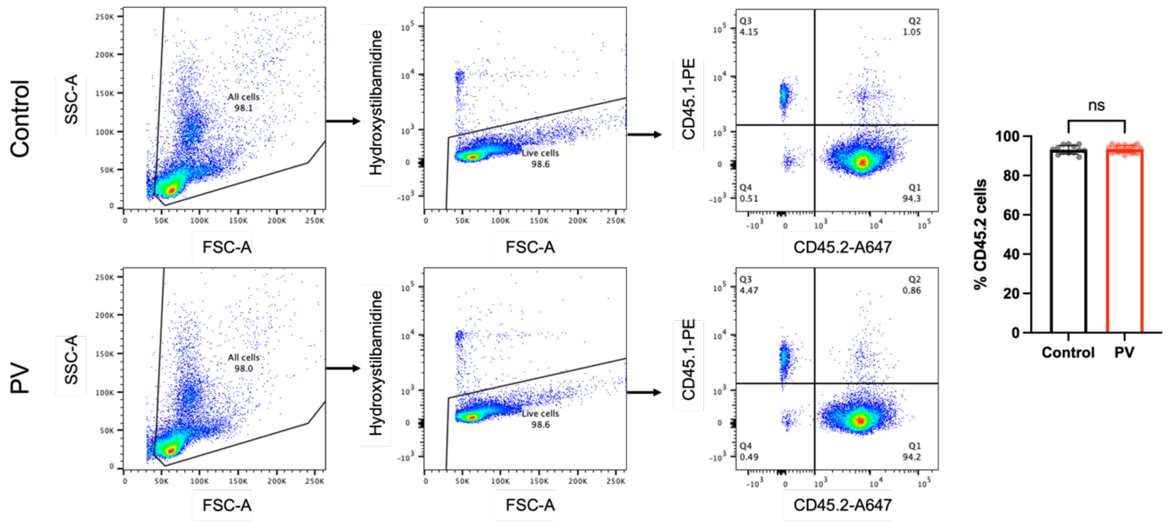

Supplemental Figure 2 - Bone marrow transplanted mice are highly chimeric. Nucleated blood cells were stained with CD45.1-PE, CD45.2-A647 and the nucleic acid stain Hydroxystilbamidine. Chimerism was determined as the percentage of CD45.2 cells by flow assisted cell sorting. Left plot, whole cells gated from fragmented cells. Middle plot, live cells gated on absence of Hydroxystilbamidine expression. Right plot, expression of CD45.1 and CD45.2 on live cells. Chimerism for control (black) and PV (red) animals is shown. N=13 control/20 PV. Unpaired 2-tailed t-test with Welch's correction. ns = non-significant

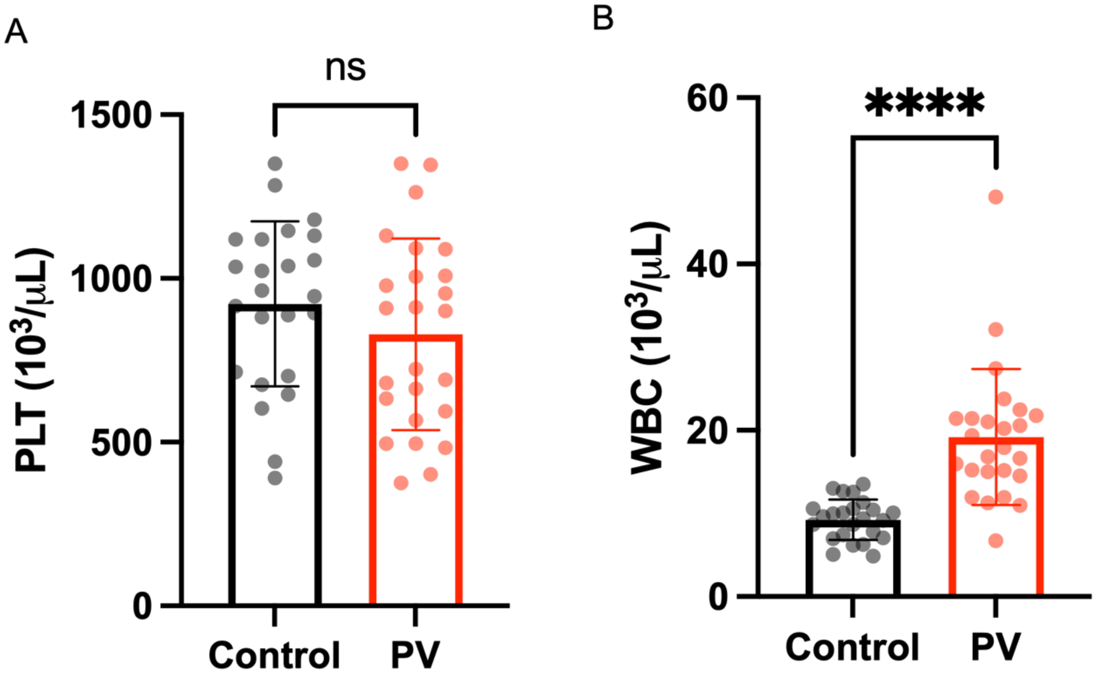

Supplemental Figure 3 – Platelet and white cell counts in control and PV mice. (A) Platelet counts (PLT); (B) white cell count (WBC) determined by automated hemocytometer. N=24 control/25 PV. (A) Unpaired 2-tailed t-test with Welch's correction or (B) Mann-Whitney test. ****$p \leq 0.0001$; ns = non-significant

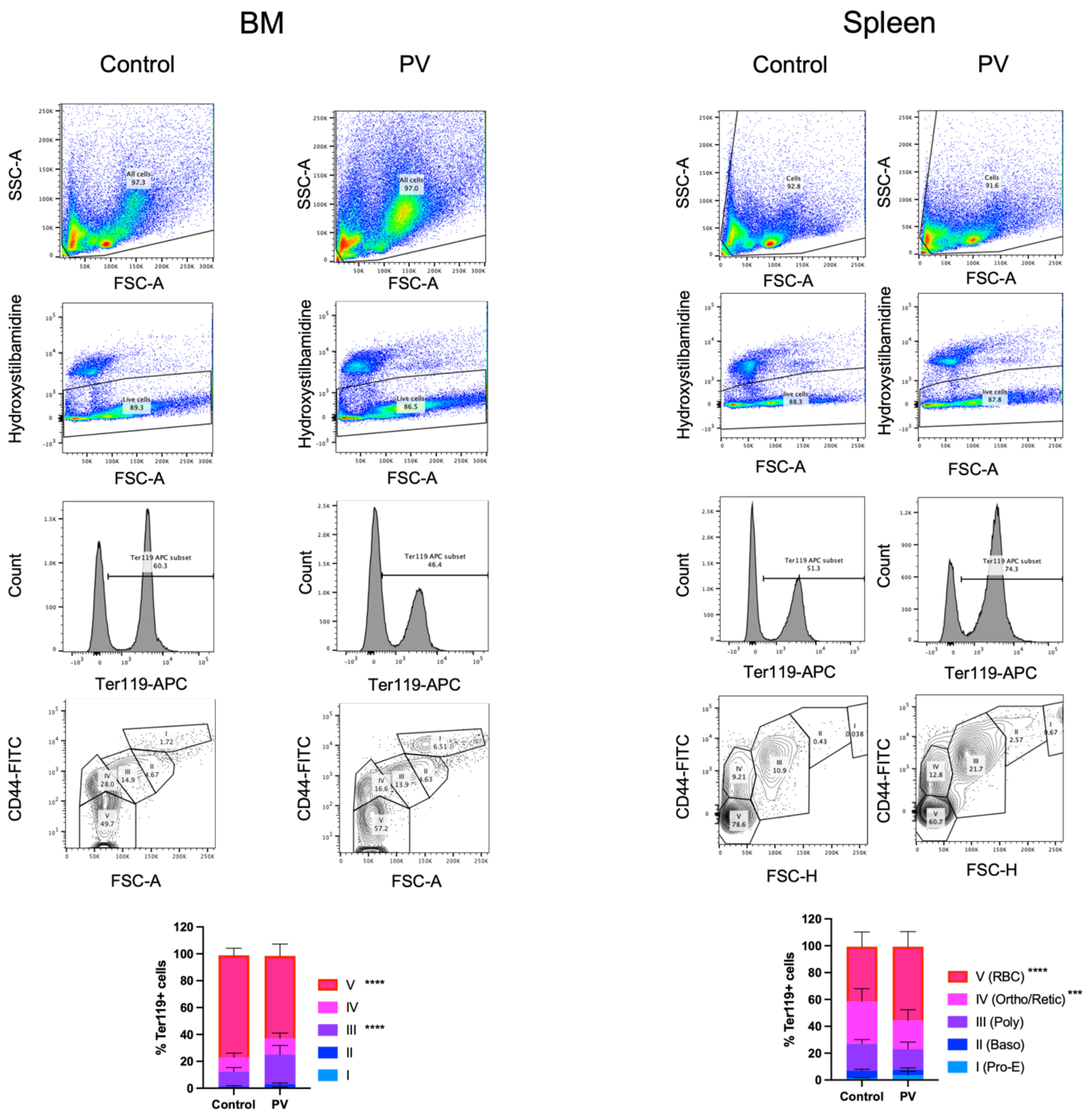

Supplemental Figure 4 – Terminal erythropoiesis is altered in the bone narrow and spleen of PV mice. Representative flow cytometry plots showing CD44 expression against Forward/Side Scatter Area (FSC-A) of Ter119 expressing (Ter119+) bone marrow (left hand side) or spleen (right hand side) cells of Control and PV mice. Based on CD44 expression and FSC-A, cells were gated into 5 distinct populations: I – proerythroblast (Pro-E), II – basophilic erythroblasts (Baso), III – polychromatic erythroblasts (Poly), IV – orthochromatic erythroblasts and reticulocytes (Ortho/Retic), and V – red blood cells (RBC). N=24 control /

25 PV bone marrow and 11 control / 8 PV spleen. 2-Way ANOVA with Šídák's correction for multiple comparisons. *** p≤ 0.001; ****p≤ 0.0001

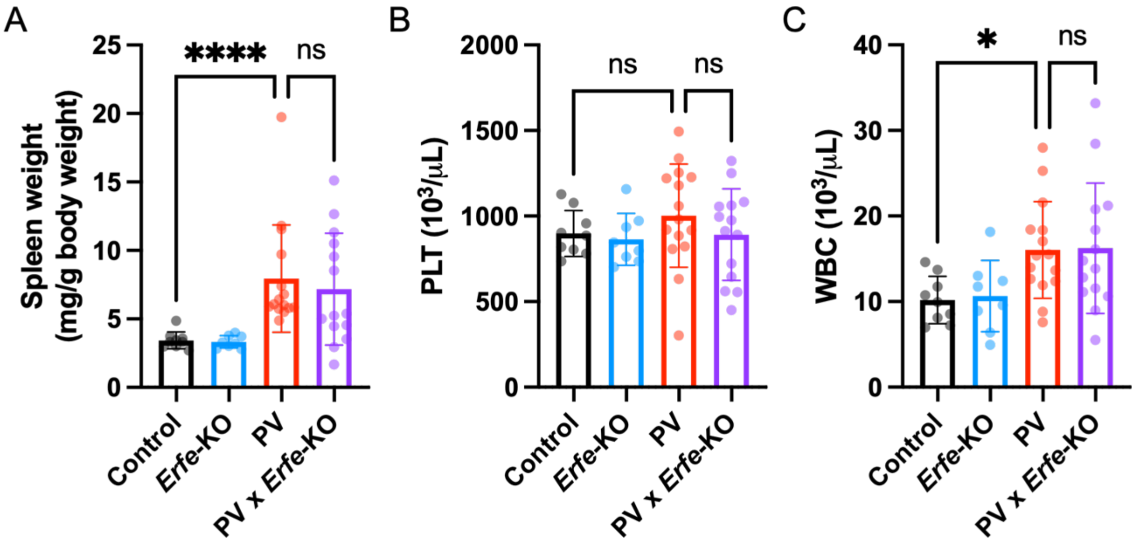

Supplemental Figure 5 – Erythroferrone deletion has no effect on spleen weight and non-erythroid haematological lineages in PV mice. (A) Spleen weight, (B) platelet count (PLT) and (C) leukocyte count (WBC) of control (black), *Erfe*-KO (blue), PV (red) and PV x *Erfe*-KO (purple) mice. N=9 Control/8 *Erfe*-KO/15 PV/14 PV x *Erfe*-KO. Kruskal-Wallis test (A) or Ordinary one-way ANOVA (B-C). **p<0.01; ****p<0.0001; ns = non-significant.

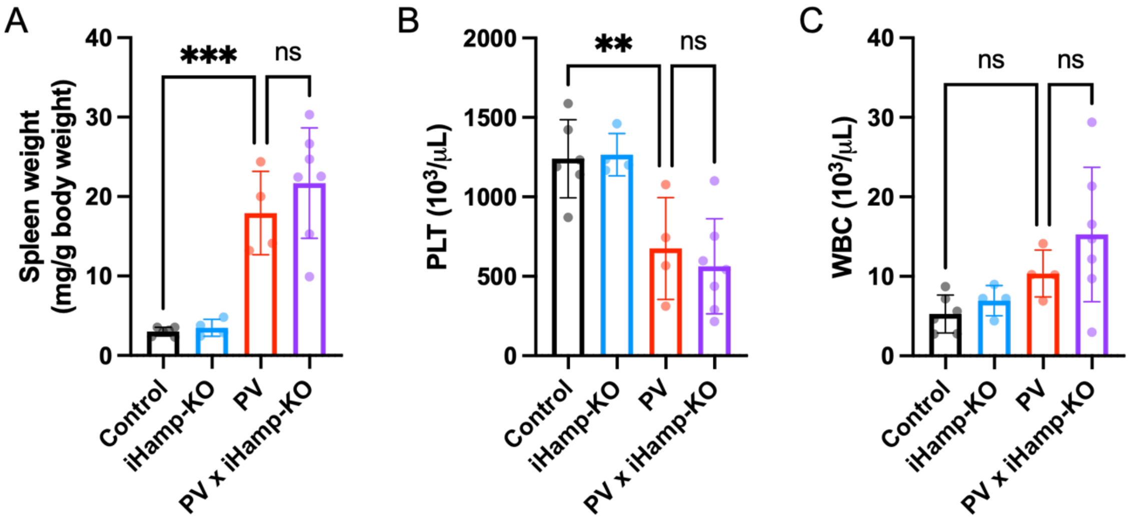

Supplemental Figure 6 – Hepcidin deletion has no effect on spleen weight and non-erythroid haematological lineages in PV mice. (A) Spleen weight, (B) platelet count (PLT) and (C) leukocyte count (WBC) of control (black), iHamp-KO (blue), PV (red) and PV x iHamp-KO (purple) mice. N=6 Control/4 iHamp-KO/4 PV/7 PV x iHamp-KO. Ordinary one-way ANOVA. **$p<0.01$; ***$p<0.001$; ns = non-significant.

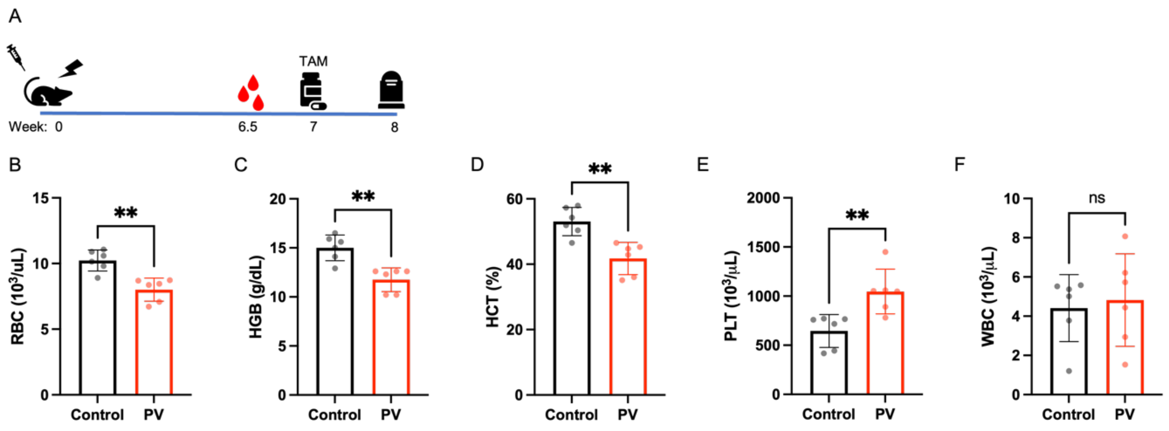

Supplemental Figure 7 – PV mice have myeloproliferative phenotype 1 week after tamoxifen induction of *Jak2*-V617F allele. (A) Schematic of experimental setup. (B) Red blood cell count (RBC), (C) hemoglobin (HBG), (D) haematocrit (HCT), (E) platelet count (PLT) and (F) white blood cell count *WBC) of control (black) and PV (red) mice determined by automated haemocytometer. N=6. Mann-Whitney test (C, E, F) or Unpaired 2-tailed t-test with Welch's correction (B, D). **$p<0.01$, ns = non-significant

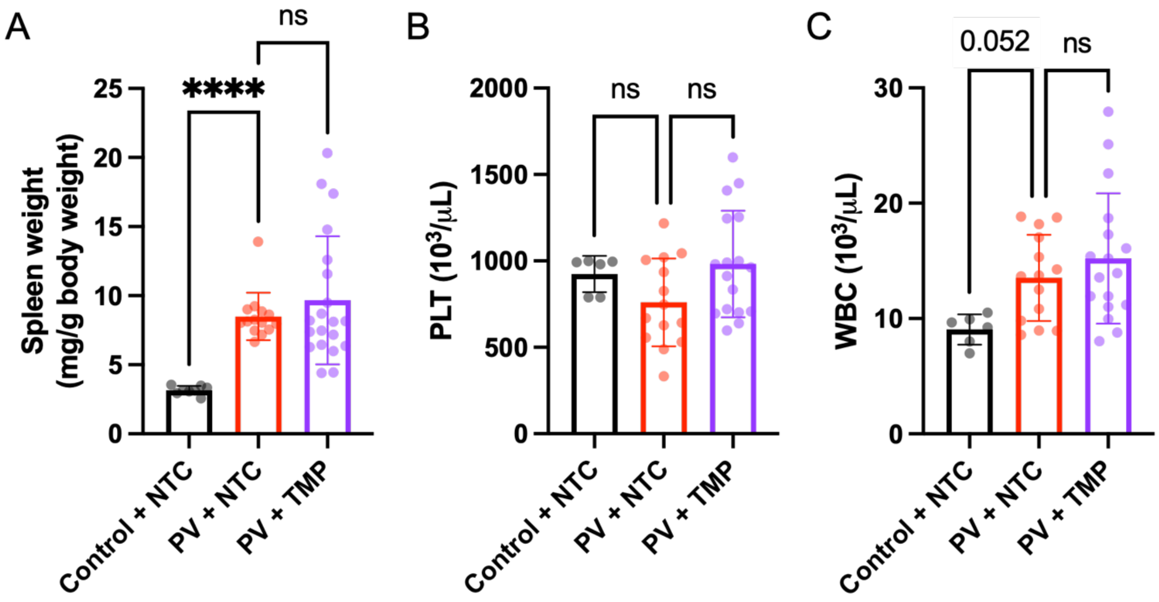

Supplemental Figure 8 – Spleen weights, platelet and white cell counts in mice treated with non-targeting control (NTC) or *TMPRSS6* (TMP) siRNA. (A) spleen weight; N=8 control+NTC/14 PV+NTC/20 PV+TMP. (B) Platelet counts (PLT) and (C) white cell count (WBC) determined by automated hemocytometer. N=6 control+NTC/14 PV+NTC/17 PV+TMP. (A, B) Kruskal-Wallis Test or (C) Oridnary One-Way ANOVA. ** $p < 0.01$; ****$p < 0.0001$; ns = non-significant

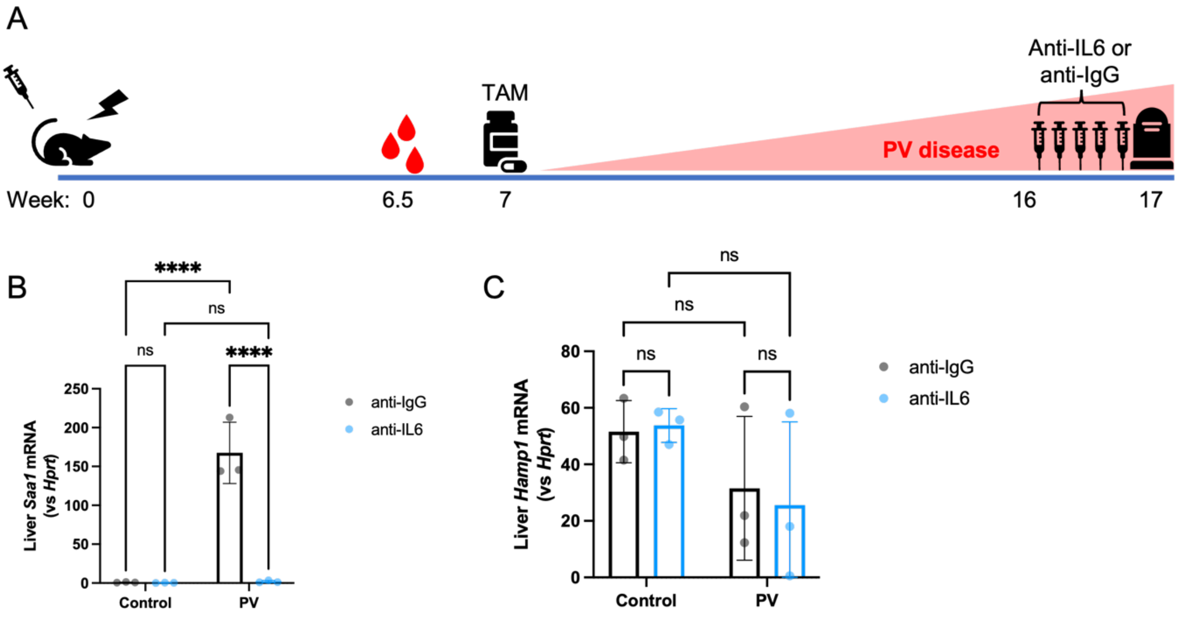

Supplemental Figure 9 – Daily anti-IL6 treatment does not affect hepcidin expression in PV mice. (A) Schematic of experimental setup – bone marrow transplanted mice were injected with anti-IL6 or anti-IgG daily for 5 days and culled 2 hours after final injection. (B) Serum amyloid A1 mRNA (*Saa1*) and (C) hepcidin mRNA (*Hamp1*) determined by qRT-PCR of liver samples. N=3. 2-Way ANOVA with Tukey's correction for multiple comparisons. ****p<0.0001, ns = non-significant

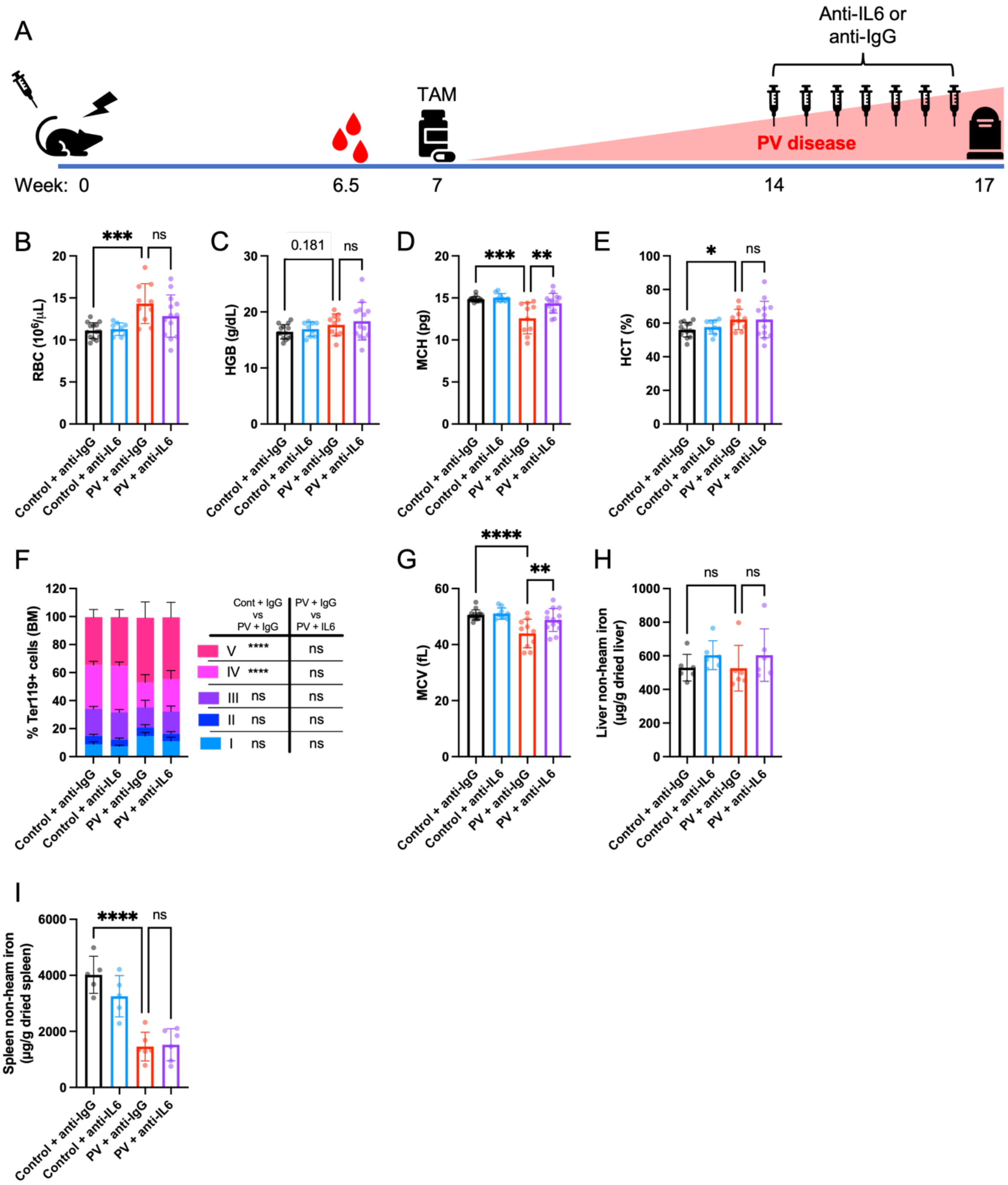

Supplemental Figure 10 – Erythropoiesis and iron homeostasis are unchanged in PV mice treated with anti-IL6. (A) Schematic of experimental setup – bone marrow transplanted mice were injected with anti-IL6 or anti-IgG every 3 days for a total of 7 injections and culled 3 days after final injection. (B) red blood cells (RBC); (C) hemoglobin (HGB); (D) mean corpuscular hemoglobin (MCH), (E) haematocrit (HCT); (F) erythropoiesis analysed by flow

cytometry of Ter119+ bone marrow cells; (G) mean corpuscular volume (MCV); (H) liver non-haem iron content; (I) spleen non-haem iron content of control or PV mice treated with anti-IL6 or anti-IgG antibodies. (B-E and G) N = 14 Control +anti-IgG/9 Control + anti-IL6/10 PV + anti-IgG/13 PV + anti-IL6. (F) N = 10 Control +anti-IgG/5 Control + anti-IL6/9 PV + anti-IgG/10 PV + anti-IL6. (H-I) N=6. Ordinary one-way ANOVA (B-C, E, G, I), Kruskal-Wallis test (D, H) or Two-way ANOVA with Tukey's correction for multiple comparisons. *p<0.05; **p<0.01; ***p<0.001; ****p<0.0001; ns – non-significant.

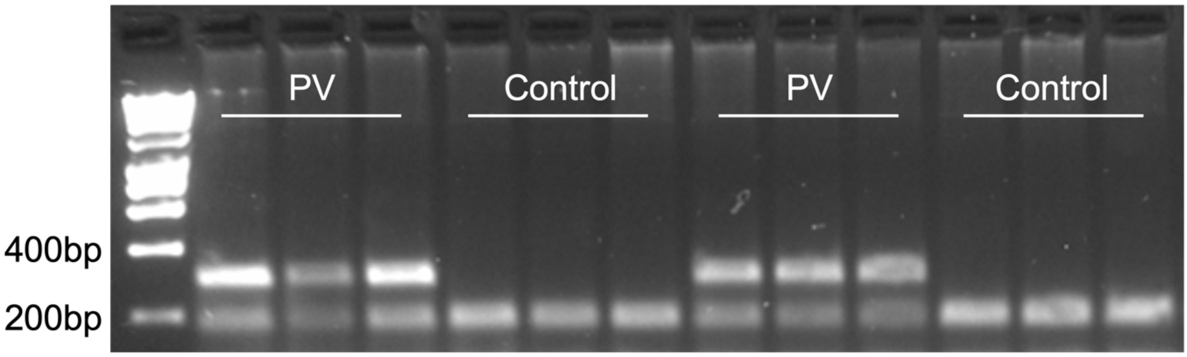

Supplemental Figure 11 – Tamoxifen treatment causes expression of Jak-V617F allele in PV mice. DNA from whole bone marrow lysates taken from PV or control mice 10 weeks after administration of tamoxifen was used for PCR to detect expression of the wildtype Jak2 allele (186bp) and the recombined Jak2-V617F allele (297bp).

References

1. Bycroft C, Freeman C, Petkova D, et al. The UK Biobank resource with deep phenotyping and genomic data. *Nature*. 2018;562(7726):203-209.
2. Astle WJ, Elding H, Jiang T, et al. The Allelic Landscape of Human Blood Cell Trait Variation and Links to Common Complex Disease. *Cell*. 2016;167(5):1415-1429.e1419.
3. Seibler J, Zevnik B, Küter-Luks B, et al. Rapid generation of inducible mouse mutants. *Nucleic Acids Res*. 2003;31(4):e12.
4. Chen K, Liu J, Heck S, Chasis JA, An X, Mohandas N. Resolving the distinct stages in erythroid differentiation based on dynamic changes in membrane protein expression during erythropoiesis. *Proceedings of the National Academy of Sciences of the United States of America*. 2009;106(41):17413-17418.
5. Liao Y, Smyth GK, Shi W. The R package Rsubread is easier, faster, cheaper and better for alignment and quantification of RNA sequencing reads. *Nucleic Acids Research*. 2019;47(8):e47-e47.
6. Liao Y, Smyth GK, Shi W. featureCounts: an efficient general purpose program for assigning sequence reads to genomic features. *Bioinformatics*. 2014;30(7):923-930.
7. Frankish A, Diekhans M, Ferreira A-M, et al. GENCODE reference annotation for the human and mouse genomes. *Nucleic Acids Research*. 2018;47(D1):D766-D773.
8. Ritchie ME, Phipson B, Wu D, et al. limma powers differential expression analyses for RNA-sequencing and microarray studies. *Nucleic Acids Research*. 2015;43(7):e47-e47.
9. Phipson B, Lee S, Majewski IJ, Alexander WS, Smyth GK. ROBUST HYPERPARAMETER ESTIMATION PROTECTS AGAINST HYPERVARIABLE GENES AND IMPROVES POWER TO DETECT DIFFERENTIAL EXPRESSION. *Ann Appl Stat*. 2016;10(2):946-963.
10. Robinson MD, McCarthy DJ, Smyth GK. edgeR: a Bioconductor package for differential expression analysis of digital gene expression data. *Bioinformatics*. 2010;26(1):139-140.
11. Robinson MD, Oshlack A. A scaling normalization method for differential expression analysis of RNA-seq data. *Genome Biology*. 2010;11(3):R25.
12. Law CW, Chen Y, Shi W, Smyth GK. voom: Precision weights unlock linear model analysis tools for RNA-seq read counts. *Genome Biol*. 2014;15(2):R29.
13. Ashburner M, Ball CA, Blake JA, et al. Gene Ontology: tool for the unification of biology. *Nature Genetics*. 2000;25(1):25-29.
14. The Gene Ontology Consortium. Expansion of the Gene Ontology knowledgebase and resources. *Nucleic Acids Research*. 2016;45(D1):D331-D338.
15. Kanehisa M, Furumichi M, Tanabe M, Sato Y, Morishima K. KEGG: new perspectives on genomes, pathways, diseases and drugs. *Nucleic Acids Res*. 2017;45(D1):D353-d361.
16. Kanehisa M, Goto S. KEGG: kyoto encyclopedia of genes and genomes. *Nucleic Acids Res*. 2000;28(1):27-30.
17. Kanehisa M, Sato Y, Kawashima M, Furumichi M, Tanabe M. KEGG as a reference resource for gene and protein annotation. *Nucleic Acids Research*. 2015;44(D1):D457-D462.
18. Subramanian A, Tamayo P, Mootha VK, et al. Gene set enrichment analysis: A knowledge-based approach for interpreting genome-wide expression profiles. *Proceedings of the National Academy of Sciences*. 2005;102(43):15545.
19. Liberzon A, Birger C, Thorvaldsdóttir H, Ghandi M, Mesirov JP, Tamayo P. The Molecular Signatures Database (MSigDB) hallmark gene set collection. *Cell Syst*. 2015;1(6):417-425.

20. Pasricha SR, Lim PJ, Duarte TL, et al. Hepcidin is regulated by promoter-associated histone acetylation and HDAC3. *Nat Commun*. 2017;8(1):403.
